# Loss of Dead end1 induces testicular teratomas from primordial germ cells that failed to undergo sexual differentiation in embryonic testes

**DOI:** 10.1038/s41598-023-33706-x

**Published:** 2023-04-19

**Authors:** Atsuki Imai, Kazuya Matsuda, Yuki Niimi, Atsushi Suzuki

**Affiliations:** 1 Division of Materials Science and Chemical Engineering, Graduate School of Engineering, Yokohama National University, Yokohama, Kanagawa Japan; 2 Division of Materials Science and Chemical Engineering, Faculty of Engineering, Yokohama National University, Yokohama, Kanagawa Japan; 3 Present Address: Research & Development Group, Center for Exploratory Research, Hitachi, Ltd., Kobe, Hyogo Japan

**Keywords:** Germline development, Pluripotency, Cancer stem cells

## Abstract

Spontaneous testicular teratomas (STTs) are tumours comprising a diverse array of cell and tissue types, which are derived from pluripotent stem-like cells called embryonal carcinoma cells (ECCs). Although mouse ECCs originate from primordial germ cells (PGCs) in embryonic testes, the molecular basis underlying ECC development remains unclear. This study shows that the conditional deletion of mouse Dead end1 (*Dnd1*) from migrating PGCs leads to STT development. In *Dnd1*-conditional knockout (*Dnd1*-cKO) embryos, PGCs colonise the embryonic testes but fail to undergo sexual differentiation; subsequently, ECCs develop from a portion of the PGCs. Transcriptomic analyses reveal that PGCs not only fail to undergo sexual differentiation but are also prone to transformation into ECCs by upregulating the expression of marker genes for primed pluripotency in the testes of *Dnd1*-cKO embryos. Thus, our results clarify the role of *Dnd1* in developing STTs and developmental process of ECC from PGC, providing novel insights into pathogenic mechanisms of STTs.

## Introduction

Testicular teratomas are tumours composed of a diverse array of cell and tissue types derived from all three germ layers, including erythrocytes, adipocytes, cartilage, muscle, hair, and glandular tissue, as well as a cluster of highly proliferative and pluripotent stem-like cells called embryonal carcinoma cells (ECCs). At the onset of spontaneous testicular teratomas (STTs), primordial germ cells (PGCs) transform into ECCs in embryonic testes and subsequently differentiate into various types of embryonic and adult cells constituting the tumours. In mice, the incidence rate of STTs varies according to the genetic background; the 129 family of inbred mouse strains causes STTs at a probability of 1–7% depending on the subline, whereas other strains, such as C57BL/6J, LTXBJ, and C3H/HeJ, rarely develop STTs^1–4^. Previous studies have identified several genes and genomic loci associated with high susceptibility to STTs in the genetic background of 129 strains^5–12^ and have attempted to analyse the function of these genetic factors in the development of STTs^13,14^. Consequently, these analyses recently suggested that ECCs developed from PGCs that failed to undergo sexual differentiation and retained early PGC characteristics even after colonising the embryonic testes^15^. However, the molecular mechanisms underlying the transformation of PGC to ECC remain unclear.

Dead end1 (*Dnd1*) encodes an RNA-binding protein specifically expressed in PGCs at embryonic stages. The expression of DND1 begins immediately after PGC development, continues during the migration and colonisation of the embryonic testes, and becomes restricted to spermatogonia in adult testes after birth, whereas its expression gradually decreases after colonisation of the embryonic ovary, as embryonic development progresses, and disappears during oogenesis^16,17^. In 2005, *Dnd1* was identified as the gene responsible for a spontaneous mutation called *Ter*^5^, isolated in 1973 as a mutation that increased STT incidence to 17% and 94% in the heterozygotes and homozygotes, respectively, in the 129/Sv genetic background^3^. *Ter* was a single nucleotide polymorphism that generated a premature stop codon in the third exon of *Dnd1*, which is presumed to result in a null mutation of *Dnd1* due to nonsense-mediated mRNA decay. Therefore, *Dnd1* is a key to understanding the mechanisms underlying STT development. However, as STTs predominantly occur in the 129 strain genetic background, not only loss of *Dnd1* but also unidentified genetic factors specific to the 129 strain family of mice are essential for STT development. In addition, the number of PGCs drastically decreases during migration and gonad colonisation owing to apoptotic cell death^18^ and some of the remaining PGCs transform to ECCs in the *Dnd1*^*Ter/Ter*^ embryonic testes, suggesting that secondary effects of *Dnd1*-loss might cause ECC development in the 129 strain genetic background. Therefore, it is challenging to clarify the role of *Dnd1* in the mechanisms of ECC development using *Ter* mutant.

We have previously shown that DND1 directly interacts with another RNA-binding protein, NANOS2, in PGCs colonising the embryonic testes and suppresses the expression of target genes by recruiting the CCR4-NOT deadenylase complex to their mRNAs through NANOS2 for degradation^16^. To elucidate the physiological significance of the DND1-NANOS2 complex, we generated a drug-inducible *Dnd1*-conditional knockout (*Dnd1*-cKO) mouse line and removed DND1 from PGCs in the embryonic testes after NANOS2 expression began. Consequently, PGCs failed to undergo sexual differentiation and were gradually lost by apoptotic cell death, even in the presence of NANOS2, resulting in small testes with no germ cells in the seminiferous tubules, as well as the testes of *Nanos2* mutant mice. Therefore, we concluded that DND1 is an essential partner of NANOS2 to regulate the sexual differentiation of PGCs in embryonic testes. In addition, we conducted a functional analysis of DND1 in the spermatogonia of adult testes using *Dnd1*-cKO mice and showed that DND1 is required for the maintenance of undifferentiated and differentiating spermatogonia^19^. Thus, DND1 plays a critical role in various germline development steps. However, the physiological significance of DND1 expression in early PGCs remains unclear.

This study aimed to investigate the effect of the loss of *Dnd1* in early PGCs and analyse the transcriptomic changes in PGCs to elucidate the mechanism underlying the transformation of PGC to ECC. Our results provide new insights on the developmental mechanisms of ECC from PGC.

## Results

### *Dnd1* deletion in migrating PGCs induces spontaneous testicular teratomas

**Figure 1:**
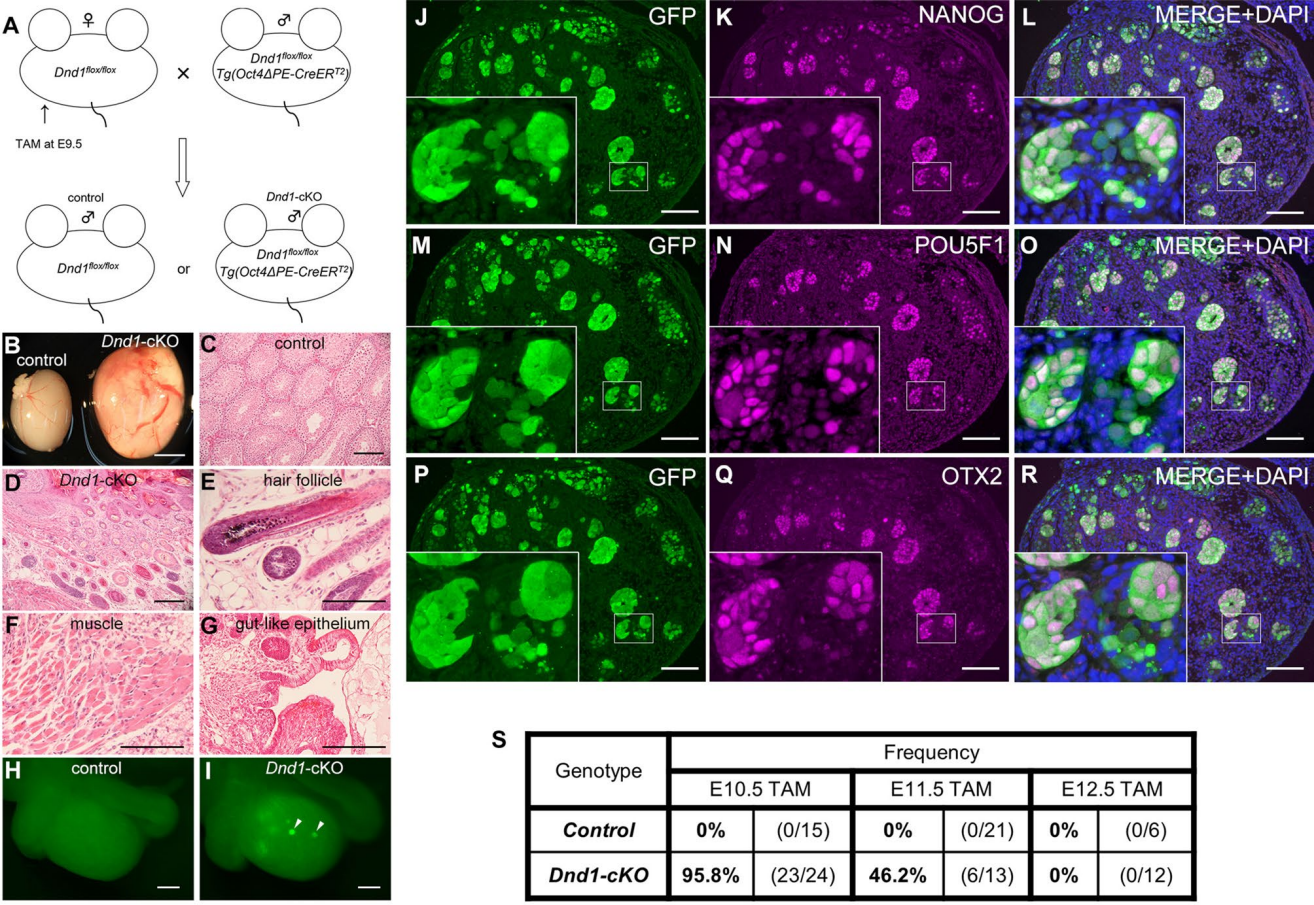
*Dnd1* deletion in migrating PGCs causes testicular teratomas. (**A**), Mating strategy for the generation of conditional knockout mice of *Dnd1*. (**B**), Comparison of testis size between 4-week-old littermates of control (*Dnd1*^*flox/flox*^) and *Dnd1*-cKO (*Dnd1*^*flox/flox*^*; Oct4ΔPE-CreER*^*T2*^) mice. (**C**–**G)**, Testis sections from 4-week-old control (**C**) and *Dnd1*-cKO (**D**–**G**) mice were stained with haematoxylin and eosin. Hair follicles, muscles, and gut-like epithelium are observed in (**E**, **F**), and (**G**), respectively. (**H**,** I**), Testis from control (**H**) and *Dnd1*-cKO (**I**) embryos with *Nanog-GFP* transgene at E16.5, under a fluorescence microscope. The arrowheads in (**I**) indicate GFP-positive foci. (**J**–**R**), Testis sections from E16.5 *Dnd1*-cKO embryos with *Nanog-GFP* transgene were immunostained with antibodies against GFP (**J**, **M**, **P**), NANOG (**K**), POU5F1 (**N**), and OTX2 (**Q**). DNA was labelled with DAPI (blue) (**L**, **O**, **R**). Images correspond to one of *n* = 3 biological replicates. **S**, The incidence of male embryos with at least one ECC colony in the testes was analysed in control and *Dnd1*-cKO embryos with *Nanog-GFP* transgene after injection of tamoxifen at E10.5, E11.5, and E12.5. Scale bars: 5 mm in (**B**), 100 μm in (**C**) and (**D**), 100 μm in (**E**–**G**), 200 μm in (**H**) and (**I**), and 100 μm in (**J**–**R**).

To examine whether DND1 plays a physiological role in the development of PGCs before sexual differentiation, we introduced the *Oct4ΔPE-CreER*^*T2*^ transgene into *Dnd1*^*flox/flox*^ mice and administered tamoxifen to pregnant mothers at embryonic day (E) 9.5, to delete DND1 from PGCs at migrating stages (Fig. 1A). We then analysed the male offspring after birth. Notably, we detected larger testes in 4-week-old *Dnd1*-cKO mice than those in control mice (Fig. 1B). Section staining analysis revealed that the control testes showed normal spermatogenesis, whereas the *Dnd1*-cKO testes contained various tissues and cell types (Fig. 1C, D). We found tissue structures derived from all three germ layers, including hair follicles from the ectoderm, muscles from the mesoderm, and gut-like epithelium from the endoderm (Fig. 1E–G), indicating that teratomas developed in the testes.

To analyse the development of STTs at earlier stages, we visualised the onset of STTs by labelling tumour precursors known as embryonal carcinoma cells (ECCs). A reporter gene expressing GFP under the control of a pluripotent stem cell marker, *Nanog*, was introduced into *Dnd1*-cKO embryos, as NANOG was expressed not only in early PGCs, but also in ECCs^20,21^. This transgene showed distinct GFP-positive foci inside the *Dnd1*-cKO testes at E16.5 (Fig. 1H, I). Section immunostaining analysis revealed that these foci consisted of colony-forming cells expressing stem cell markers, such as NANOG and POU5F1, and a marker for ECCs, OTX2^13^ (Fig. 1J–R). Meanwhile, the PGCs that were negative for these markers also faintly expressed GFP. These data indicate that the colony-forming cells strongly expressing GFP are putative ECCs that generate teratomas at later stages; therefore, the *Nanog-GFP* transgene allows us to promptly detect the initiation of teratoma development in the embryonic testes.

We then examined whether the timing of *Dnd1* loss in PGCs contributed to the onset of teratoma because *Dnd1*-cKO mice did not develop STTs when tamoxifen was injected at E13.5^16^. To this end, we injected tamoxifen to foster mothers at each stage from E10.5 to E12.5 and analysed the frequency of development of the GFP-positive foci in the embryonic testes using section-immunostaining or GFP fluorescence analysis. The GFP-positive foci were observed in the testes of *Dnd1*-cKO embryos at a frequency of 95.8% when tamoxifen was injected at E10.5; however, a gradual decrease to 46.2% and 0% was detected at E11.5 and E12.5, respectively (Fig. 1S).

Since NANOS2 expression begins at E12.5^22^, these results indicate that DND1 suppresses the onset of STTs in PGCs that colonise the embryonic testes, independent of NANOS2.

### Colony-forming cells are ECCs originating from PGCs in the testes of *Dnd1*-cKO embryos

**Figure 2:**
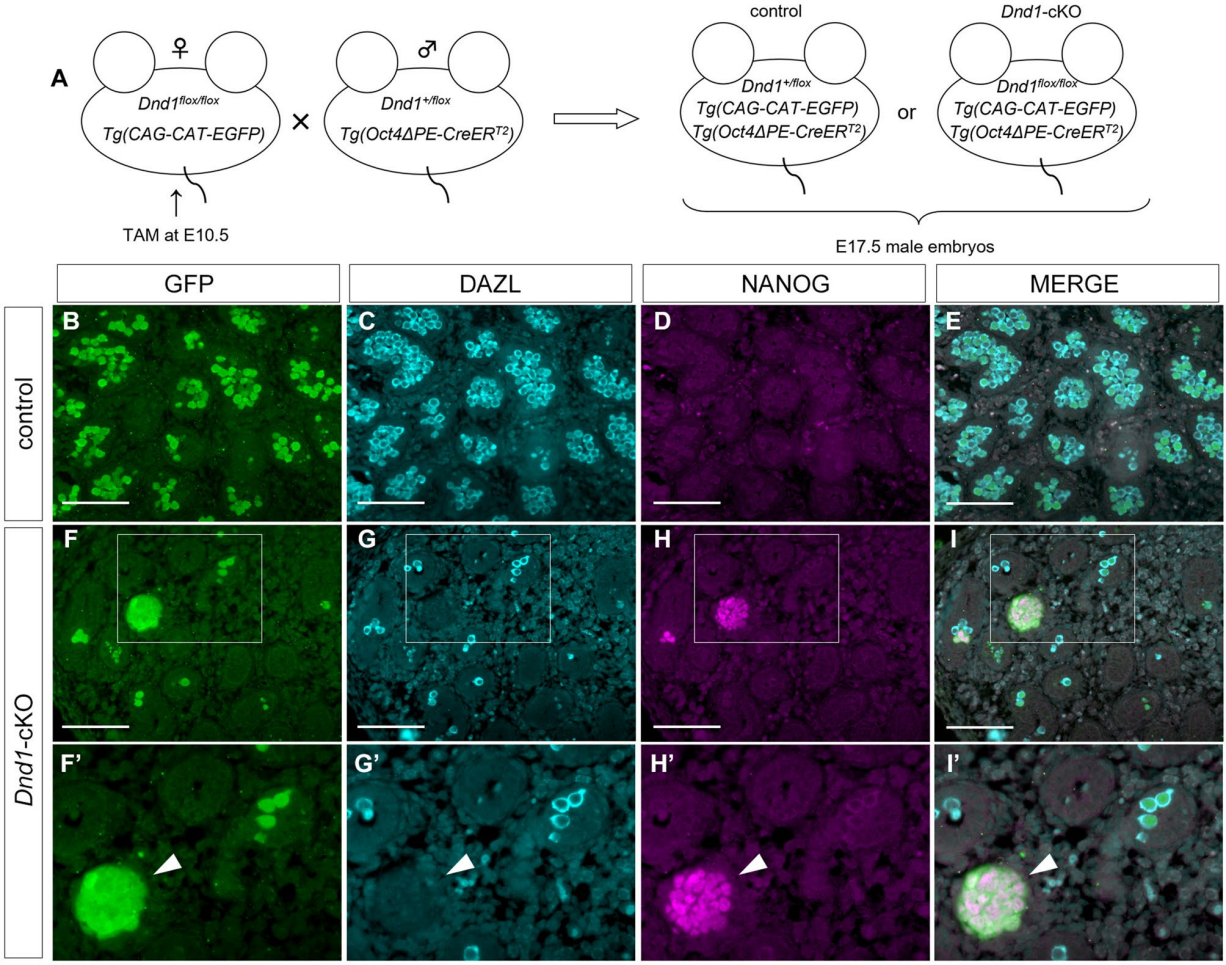
ECCs originated from PGCs in the *Dnd1*-cKO testes. (**A**), Strategy for lineage tracing of PGCs in control and *Dnd1-*cKO embryos. (**B**–**I**), Testis sections from E17.5 control (*Dnd1*^+*/flox*^*; CAG-CAT-EGFP; Oct4ΔPE-CreER*^*T2*^) and *Dnd1*-cKO (*Dnd1*^*flox/flox*^*; CAG-CAT-EGFP; Oct4ΔPE-CreER*^*T2*^) embryos were immunostained with antibodies against GFP (**B**, **F**), DAZL (**C**, **G**), and NANOG (**D**, **H**). The merged images are shown in (**E**) and (**I**). Enlarged views of the area enclosed by rectangles in (**F**, **G**, **H**), and (**I**) are shown in (**F′**, **G′**, **H′**), and (**I′**), respectively, to better visualise the expression of each protein. Images correspond to one of *n* = 4 biological replicates. Arrowheads in (**F′**, **G′**, **H′**), and (**I′**) indicate a ECC colony positive for GFP. Scale bars: 100 μm.

ECCs originate from PGCs that colonise the embryonic testes at the onset of STTs^23,24^. To confirm that the *Nanog-GFP*-positive cell foci were indeed ECCs, we examined whether the cell foci were derived from PGCs in the *Dnd1*-cKO embryonic testes. We introduced the *CAG-CAT-EGFP* transgene into *Dnd1*^*flox/flox*^ embryos with the *Oct4ΔPE-CreER*^*T2*^ transgene and traced the developmental fate of the PGCs in the absence of DND1 by injecting tamoxifen at E10.5 (Fig. 2A). As it has been reported that ECCs highly express NANOG and lose the expression of a germ cell marker deleted in azoospermia (DAZL)^13^, we conducted section-immunostaining analysis using antibodies against GFP, NANOG, and DAZL. GFP marked approximately 70% of DAZL-positive PGCs in the E17.5 control testes in this experimental system (Fig. 2B–E). In the testes of E17.5 *Dnd1*-cKO embryos, there were only a few DAZL-positive PGCs, and some NANOG-positive and DAZL-negative cell foci developed as expected (Fig. 2F–I). As the cells consisting of foci were marked by GFP, we concluded that these cells were ECCs derived from PGCs. In addition, these findings suggest that in the absence of DND1, only a fraction of PGCs transformed into ECCs, whereas most of the remaining PGCs disappeared by E17.5.

### ECCs develop from a fraction of PGCs after E16.5 in the testes of *Dnd1*-cKO embryos

**Figure 3:**
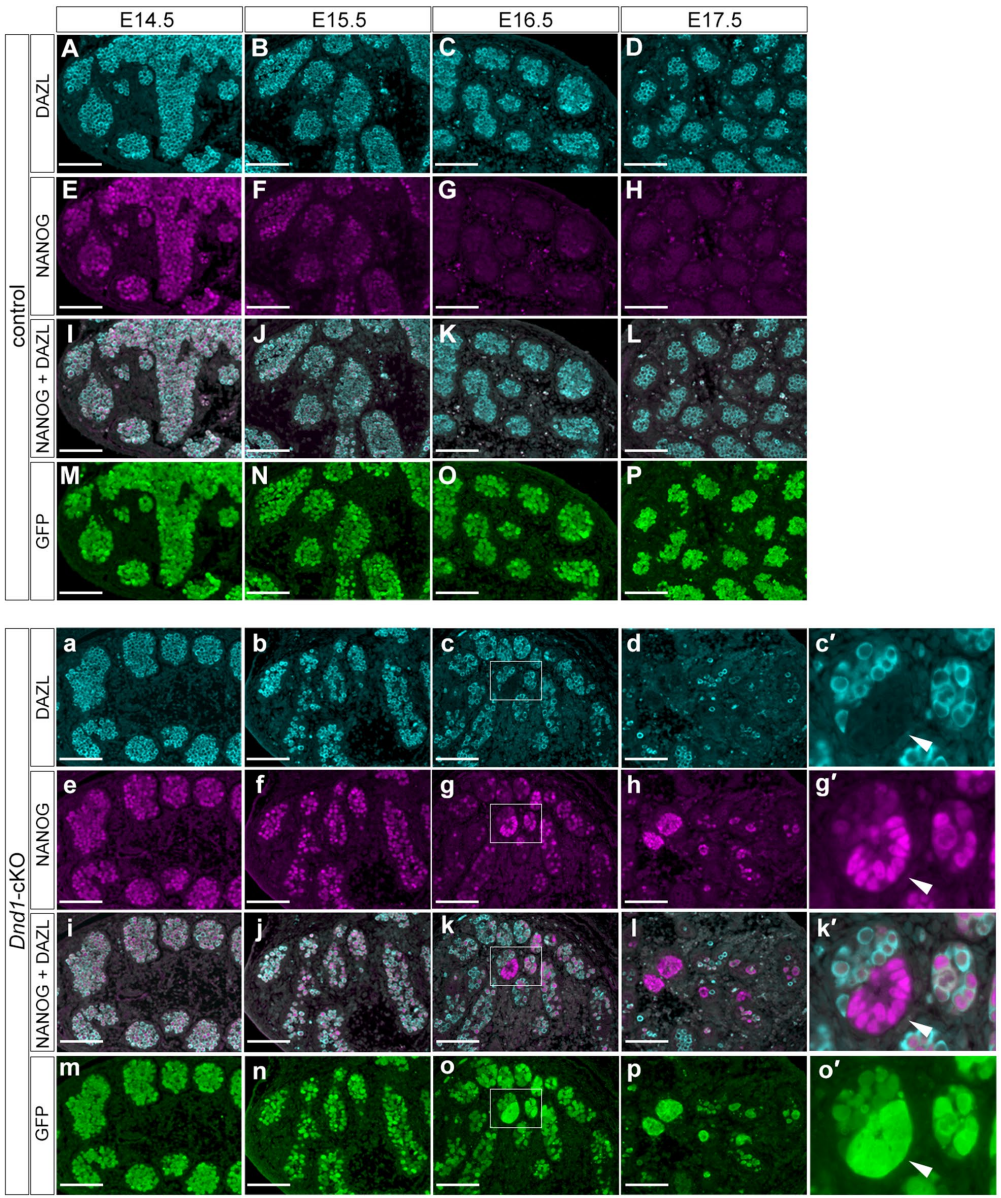
ECCs develop from a fraction of PGCs after E16.5 in *Dnd1-*cKO testes. (**A**–**p**), Testis sections from the control (*Dnd1*^*flox/flox*^*; Nanog-GFP*) (**A**–**P**) and *Dnd1*-cKO (*Dnd1*^*flox/flox*^*; Nanog-GFP; Rosa26-CreER*^*T2*^) (**a**–**p**) embryos at E14.5, E15.5, E16.5, and E17.5, were immunostained with antibodies against DAZL (**A**–**D**, **a**–**d**), NANOG (**E**–**H**, **e**–**h**), and GFP (**M**–**P**, **m**–**p**). Merged images of DAZL and NANOG are shown in (**I**–**L**) and (**i**–**l**), respectively. Enlarged views of the area enclosed by rectangles in (**c**, **g**, **k**), and (**o**) are shown in (**c′**, **g′**, **k′**), and (**o′**), respectively, to better visualise the expression of each protein. Images correspond to one of *n* > 3 biological replicates. Arrowheads in (**c′**, **g′**, **k′**), and (**o′**) indicate a ECC colony. Scale bars: 100 μm.

We next analysed when and how the ECCs developed from PGCs to further examine the onset of STTs. We used *Rosa26-CreER*^*T2*^, instead of *Oct4ΔPE-CreER*^*T2*^, to delete *Dnd1* from PGCs more efficiently and conducted section-immunostaining analysis of the testes from the control and *Dnd1*-cKO embryos using the *Nanog-GFP* transgene at E14.5, E15.5, E16.5, and E17.5 with antibodies against DAZL, NANOG, and GFP. DAZL was continuously expressed in the PGCs of the control testes at these stages, whereas NANOG expression was detected in PGCs at E14.5 but gradually decreased and almost disappeared by E16.5 (Fig. 3A–L). GFP expression was, however, maintained in the PGCs until E17.5, possibly due to the higher stability of GFP than that of NANOG (Fig. 3M–P).

We found clear differences between the testes of *Dnd1*-cKO embryos and those of littermate controls. Although there were no apparent histological differences between the control and *Dnd1*-cKO testes at E14.5, the number of PGCs began to decrease at E15.5, and most of them disappeared by E17.5 in the *Dnd1*-cKO testes, as shown by DAZL staining (Fig. 3a–d, and Fig. S1A). In contrast, the expression of NANOG was maintained at a high level in some PGCs in *Dnd1*-cKO testes at E15.5 (Fig. 3e, f, and Fig. S1B ), whereas these cells co-expressed DAZL and did not form cell foci (Fig. 3i, j). However, some small cell foci began to develop at E16.5, where NANOG was expressed at a high level, while DAZL expression was not observed (Fig. 3g, k). The number of such cell increased at E17.5 (Fig. 3h, l, and Fig. S1C). Both PGCs and ECCs were marked with GFP during these stages (Fig. 3m–p).

Since ECCs are derived from PGCs, these results indicate that only a fraction of PGCs transform into colony-forming ECCs by E16.5, acquiring a high level of NANOG expression and a loss of DAZL expression during the initiation of STTs. In contrast, the other PGCs began to decrease in number after E15.5 and almost disappeared until E17.5 in the testes of *Dnd1*-cKO embryos.

### Loss of DND1 causes accumulation of PGCs that failed to undergo sexual differentiation in embryonic testes

Next, we explored what happens to PGCs following *Dnd1* loss during ECC development. Recently, ECCs are suggested to develop from PGCs that failed to undergo sexual differentiation in embryonic testes, in which case PGCs show retention of pluripotency gene expression, downregulation of male-type gene expression, failure of mitotic arrest, and upregulation of meiotic gene expression^15,25^. Therefore, we examined whether PGCs successfully underwent male-type differentiation in the testes of *Dnd1*-cKO embryos expressing the *Nanog-GFP* transgene. As the retention of pluripotency gene expression was already shown by NANOG expression (Fig. 3E–G, e–g, and S1B), the expression of NANOS2 was first analysed, because NANOS2 is a marker for male-type differentiation of PGCs; the loss of this protein causes failure of sexual differentiation, followed by apoptotic cell death^25^.

**Figure 4:**
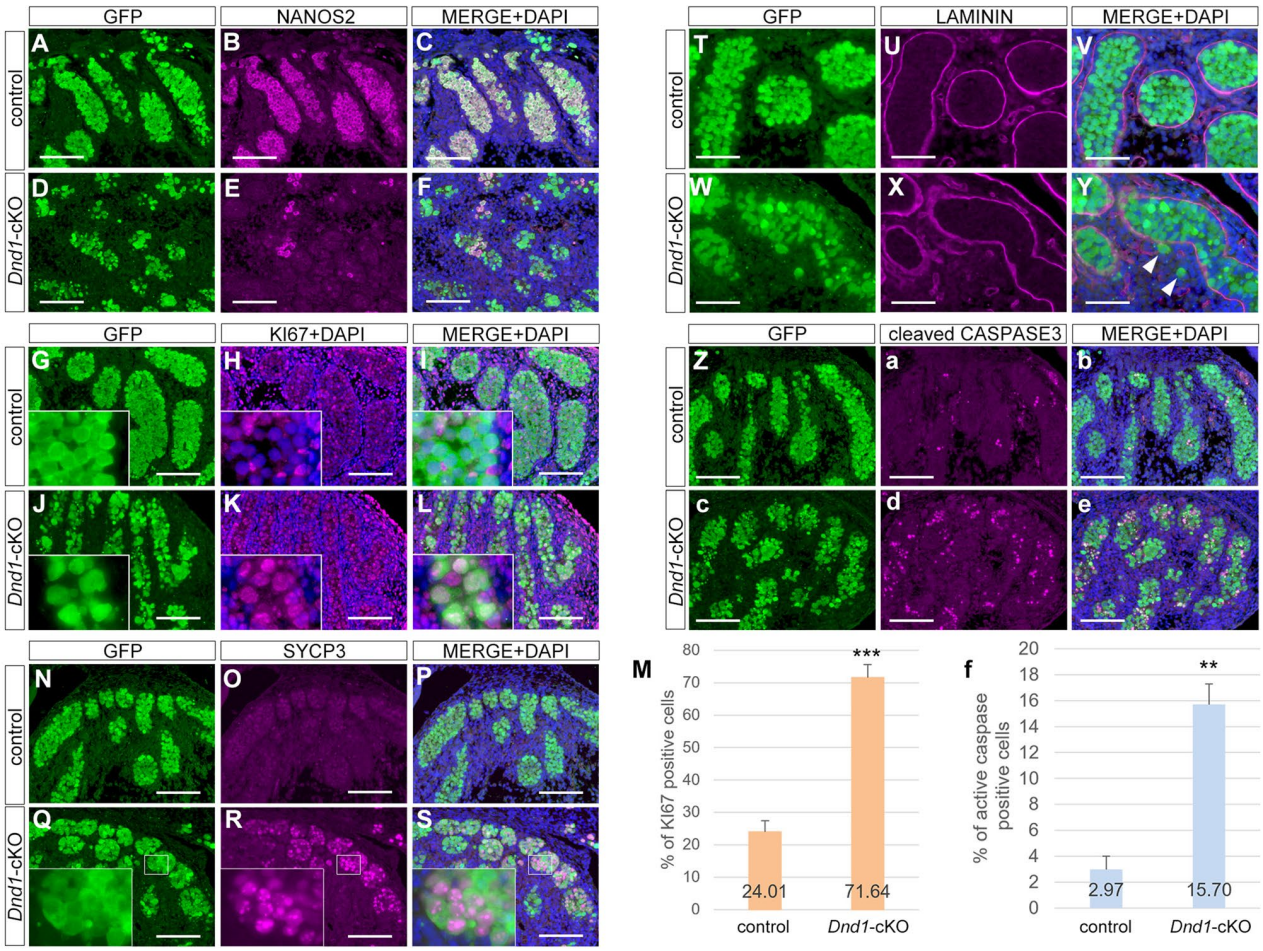
Accumulation of PGCs that failed sexual differentiation in *Dnd1-*cKO testes. **A**–**L and N–e**, Testis sections from E15.5 control (*Dnd1*^*flox/flox*^*; Nanog-GFP*) (**A**–**C**, **G**–**I**, **N**–**P**, **T**–**V**, and **Z**–**b**) and *Dnd1*-cKO (*Dnd1*^*flox/flox*^*; Nanog-GFP; Rosa26-CreER*^*T2*^) (**D**–**F**, **J**–**L**, **Q**–**S**, **W**–**Y**, and **c**–**e**) embryos were immunostained with antibodies against GFP (**A**, **D**, **G**, **J**, **N**, **Q**, **T**, **W**, **Z**, and **c**), NANOS2 (**B**, **E**), KI67 (**H**, **K**), SYCP3 (**O**, **R**), LAMININ (**U**, **X**), and cleaved CASPASE3 (**a**, **d**). DNA was labelled with DAPI (**C**, **F**, **I**, **L**, **P**, **S**, **V**, **Y**, **b**, and **e**). Enlarged views in (**G**–**L**) and (**Q**–**S**) are shown to better visualise KI67-positive and SYCP3-positive PGCs, respectively. Note that PGCs exhibit some aggregates of SYCP3 in the nucleus in (**R**) as previously reported^25,47^. Images correspond to one of *n* > 3 biological replicates. Arrowheads in **Y** indicate PGCs outside seminiferous tubules. Scale bars: 100 μm. (**M**, **f**), Ratios of (**M**) KI67- and (**f**) cleaved CASPASE3-positive PGCs per GFP-positive PGC in the E15.5 control and *Dnd1*-cKO testes. Three embryos were analysed for each genotype. Black bars represent the mean ± SD. **p < 0.01, ***p < 0.001 (Student’s *t*-test).

Immunostaining analysis revealed that NANOS2 was expressed in almost all PGCs in the control embryonic testes at E15.5, except for a few PGCs remaining in the mesonephros (Fig. 4A–C). In contrast, NANOS2 was rarely expressed in PGCs in the *Dnd1*-cKO embryonic testes, although a few PGCs appeared to normally express NANOS2 (Fig. 4D–F).

To examine the reason for the expression of NANOS2 in a few PGCs of the *Dnd1*-cKO embryonic testes, we analysed the expression of DND1. We detected its expression in some NANOS2-positive PGCs, indicating that *Dnd1* was retained in these cells because of the inefficiency of Cre recombinase (Supplementary Fig. S2A–H). However, we also found that some PGCs expressed considerable amounts of NANOS2 despite the lack of DND1, whereas most PGCs expressed NANOS2 at a very low level (Supplementary Fig. S2I–L). These data indicate that NANOS2 begins to be expressed even in the absence of DND1 but requires DND1 for its full expression in most PGCs. However, even if NANOS2 is expressed, PGCs are presumed to fail to undergo male-type differentiation in *Dnd1*-cKO testes because NANOS2 is not fully functional without DND1^16^. Consistent with this notion, we found several phenotypes corresponding to the failure of sexual differentiation in the PGCs of E15.5 *Dnd1*-cKO testes. The percentage of KI67-positive PGCs was increased from 24.0% in the control testes to 71.6% in the *Dnd1*-cKO testes (Fig. 4G–M), indicating that mitotic arrest was disrupted. A meiotic protein, SYCP3, was highly expressed and exhibited several nuclear aggregates (Fig. 4N–S), which may be located in the nucleoli, as accumulation of SYCP3 in the nucleoli can be a marker of meiotic progression, as previously reported^26,27^. A few PGCs were found outside the testis cords (Fig. 4T–Y). In addition, the percentage of activated CASPASE3-positive PGCs was drastically increased from 2.97% in the control testes to 15.7% in the *Dnd1*-cKO testes (Fig. 4Z, and a–f), accounting for the reduction in the PGC number in the *Dnd1*-cKO testes after E15.5. All these phenotypes were also observed in *Nanos*2-KO embryonic testes^28^, indicating that loss of DND1 in migrating PGCs causes accumulation of PGCs that are unable to undergo sexual differentiation in the embryonic testes.

### Increase in embryonic germ cell (EGC) derivation from *Dnd1*-cKO PGCs

PGCs can be reprogrammed into pluripotent stem cells, also known as EGCs, when cultured with specific cytokines, such as leukaemia inhibitory factor (LIF) and basic fibroblast growth factor (bFGF) on feeder cells expressing membrane-bound steel factor^29,30^. The efficiency of EGC derivation is the highest immediately after PGC development and gradually decreases with increasing embryonic age; eventually, PGCs lose the capacity to generate EGCs after E15.5^31,32^. It was reported that in *Dazl*-null mice, PGCs failed to undergo sexual differentiation in the embryonic testes and retained a high capacity to generate EGC compared to wild-type PGCs, even at E15.5^8^, suggesting that pluripotency is less restrictive in the PGCs failing sexual differentiation in embryonic testes. Therefore, we speculated that the PGCs also maintain a relatively high capacity to generate EGC in the testes of *Dnd1*-cKO embryos, since they fail to undergo sexual differentiation as well as the PGCs in the testes of *Dazl*-null embryos.

**Figure 5:**
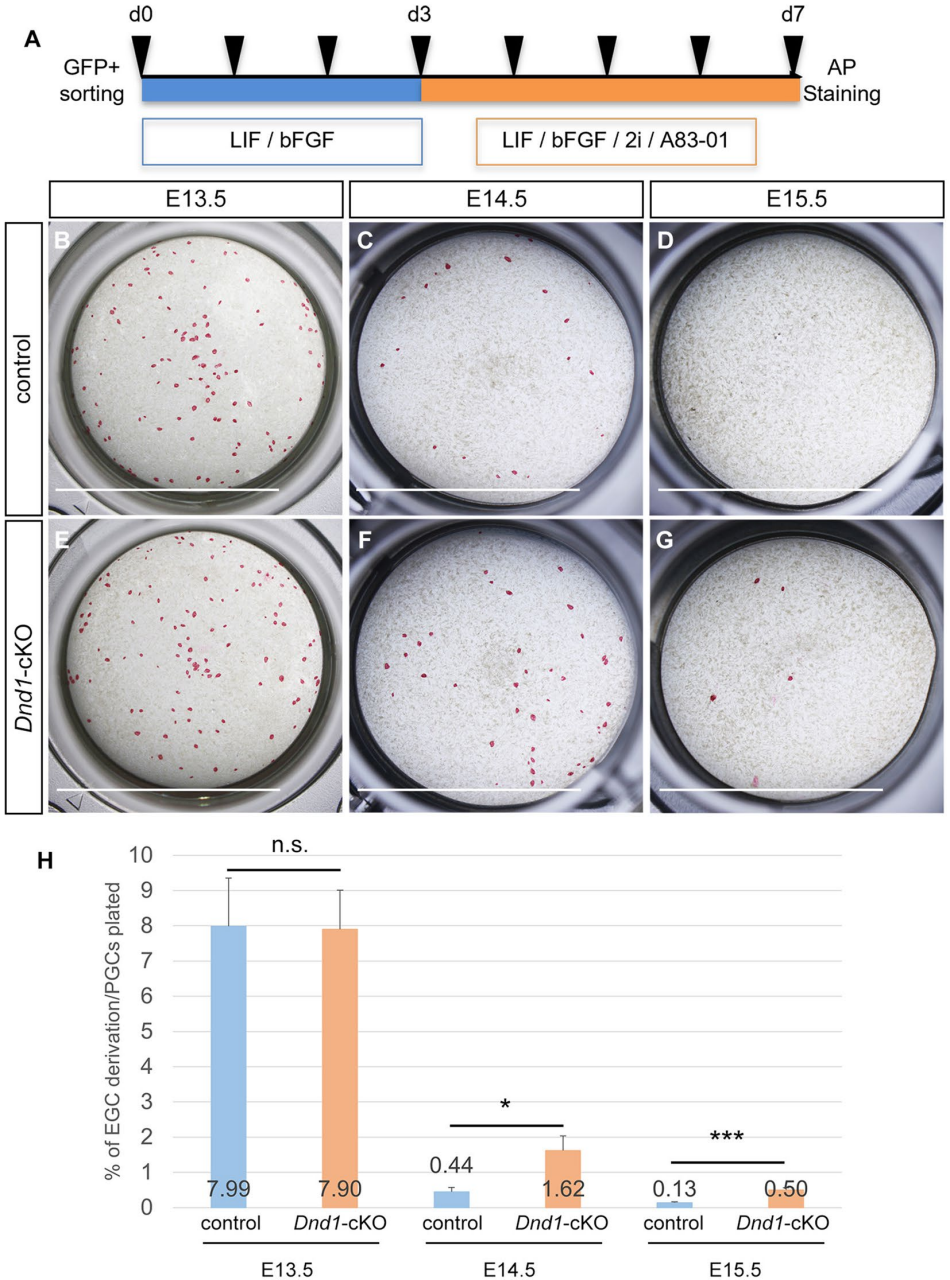
Increase in EGC formation in *Dnd1-*cKO PGCs. (**A**), Schematic representation of the culture conditions for deriving EGCs from PGCs. (**B**–**G**), Representative images of EGC colonies derived from EGFP-positive PGCs in the testes of control (*Dnd1*^*flox/flox*^*; Nanog-GFP*) (**B**–**D**) and *Dnd1*-cKO (*Dnd1*^*flox/flox*^*; Nanog-GFP; Rosa26-CreER*^*T2*^) (**E**–**G**) embryos at E13.5 (**B**, **E**), E14.5 (**C**, **F**), and E15.5 (**D**, **G**). EGC colonies were stained by alkaline phosphatase (AP) activity. Scale bars: 10 mm. (**H**), Rate of EGC derivation from EGFP-positive PGCs in the testes of control (*Dnd1*^*flox/flox*^*; Nanog-GFP*) and *Dnd1*-cKO (*Dnd1*^*flox/flox*^*; Nanog-GFP; Rosa26-CreER*^*T2*^) embryos. Black bars represent the mean ± SD. **p < 0.01, ***p < 0.001 (Student’s *t*-test).

To test this hypothesis, we attempted to derive EGCs from PGCs in the control and *Dnd1*-cKO embryos using the *Nanog-GFP* transgene. PGCs were isolated using fluorescence-activated cell sorting (FACS) (Supplementary Fig. S3A–N), plated with Sl/Sl4-m220 feeder cells expressing membrane-bound steel factor, and cultured with the medium containing LIF and bFGF as shown in Fig. 5A. The PGCs isolated from E13.5 testes of the control embryos gave rise to EGC colonies, with a mean derivation efficiency of 7.99 ± 1.37% per EGFP-positive PGCs plated; however, the efficiency drastically decreased to 0.44 ± 0.13% at E14.5, reaching 0.13 ± 0.02% at E15.5 (Fig. 5B–D, and H, control). These observations were roughly consistent with previous reports, although the efficiency was relatively high at each stage^31,32^. In contrast, PGCs from E14.5 to E15.5 testes of the *Dnd1*-cKO embryos retained a significantly higher capacity to generate EGC colonies than those of control embryos (1.62 ± 0.41% at E14.5 and 0.50 ± 0.06% at E15.5), while PGCs from E13.5 testes of the *Dnd1*-cKO embryos gave rise to EGCs with comparable derivation efficiency to those of control PGCs (7.90 ± 1.11%) (Fig. 5E–G, and H, *Dnd1*-cKO). There was no apparent difference in EGC derivation efficiency from PGCs between *Dnd1*-cKO and control female embryos during these stages (Supplementary Fig. S4A–G).

These results indicate that DND1 restricts the pluripotency of PGCs in embryonic testes after E14.5, which subsequently makes it easier for PGCs to become pluripotent in the absence of DND1.

### Transcriptomic analysis of *Dnd1*-cKO PGCs in the embryonic testes

**Figure 6:**
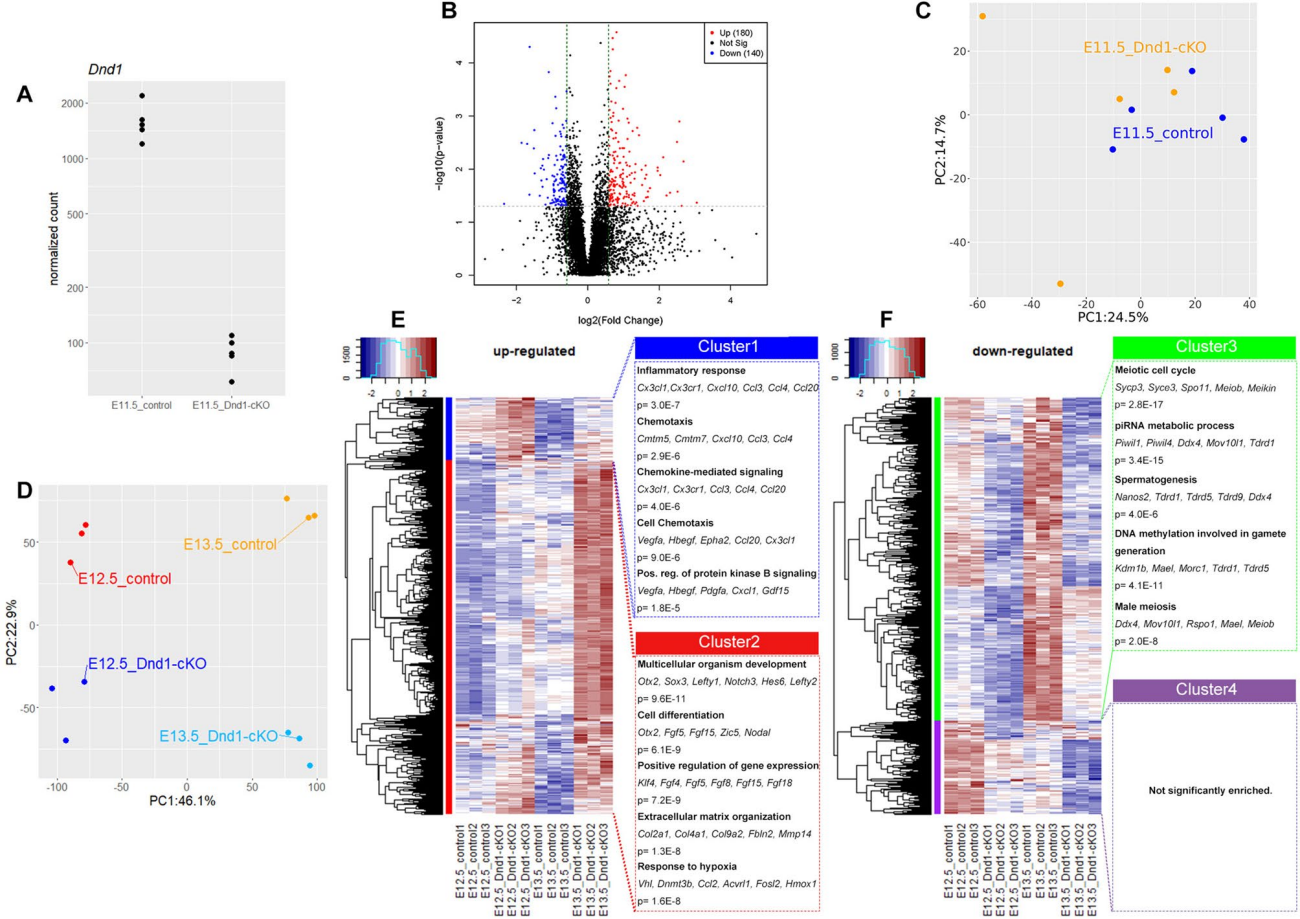
Transcriptomic analysis of PGCs in testes from the control and *Dnd1*-cKO embryos. (**A**), Expression levels of *Dnd1* transcript in each PGC from five independent control and *Dnd1*-cKO embryos are indicated by read counts in the sequence analyses. (**B**), Volcano plot of RNA-seq data of PGCs from the control and *Dnd1*-cKO embryos at E11.5. Red: *p* < 0.05, fold change > 1.5; blue: *p* < 0.05, fold change < 1.5. (**C**) PCA of the transcriptomes of PGCs from control and *Dnd1*-cKO mice at E11.5. Each dot represents a single transcriptome. Transcriptomes from five biological replicates are plotted. (**D**), PCA of the transcriptomes of PGCs in testes from control (*Dnd1*^*flox/flox*^*; Nanog-GFP*) and *Dnd1*-cKO (*Dnd1*^*flox/flox*^*; Nanog-GFP; Rosa26-CreER*^*T2*^) embryos at E12.5 and E13.5. Each dot represents a single transcriptome. Transcriptomes from three replicates were plotted. (**E**, **F**), Heatmap representation of the expression and GO enrichment of genes upregulated (**E**) and downregulated (**F**) in PGCs from *Dnd1*-cKO embryos compared to those from control embryos at E12.5 and E13.5. The heatmap was generated using the ‘heatmap.2’ function in the gplot package (ver.3.1.3, https://rdocumentation.org/packages/gplots/versions/3.1.3) after performing gene clustering using ‘dist’, ‘hclust’, and ‘cutree’ functions in the stats package (ver.3.6.2, https://rdocumentation.org/packages/stats/versions/3.6.2).

We investigated the transcriptomic changes in *Dnd1*-cKO PGCs relative to control PGCs to determine the cause of the transformation from PGC to ECC. *Dnd1*^*flox/flox*^*; Nanog-GFP* females were crossed with *Dnd1*^*flox/flox*^*; Rosa26-CreER*^*T2*^ males and injected with tamoxifen at E10.5. PGCs were then isolated from the testes of five independent *Dnd1*-cKO and control embryos by FACS at E11.5 and subjected to RNA-seq analysis, as previously described^33^. The numbers of *Dnd1* transcripts were drastically decreased in *Dnd1*-cKO PGCs compared with those in control PGCs (Fig. 6A), and 180 upregulated and 140 downregulated differentially expressed genes (DEGs) were identified in the *Dnd1*-cKO PGCs (Fig. 6B and Supplementary Table S1). However, Gene Ontology (GO) analysis revealed that GO terms were not significantly enriched in the DEGs, and principal component analysis (PCA) showed that the *Dnd1*-cKO PGC clustered closely with the control PGC (Fig. 6C), suggesting that these PGCs have relatively similar transcriptomes.

Therefore, we further analysed the transcriptomic changes by comprehensive microarray analyses using PGCs isolated from three independent male *Dnd1*-cKO and control embryos at E12.5 and E13.5. PCA analysis showed that *Dnd1*-cKO PGCs were separated from those of control at E12.5 and then progressed in opposite directions from control PGCs as the embryonic stage progress to E13.5, suggesting that the transcriptional profile in these mutant PGCs continue to diverge from the normal trajectory (Fig. 6D). To further investigate these differences, we extracted significant DEGs in *Dnd1*-cKO PGCs by using a threshold of 1.5 fold change as the cut-off value and subsequently clustered them (Fig. 6E, F, and Supplementary Table S2). Clusters 1 and 2 contained upregulated DEGs, in which the expression of the DEGs in cluster 1 was downregulated from E12.5 to E13.5, whereas the DEGs in cluster 2 were upregulated at E13.5, compared to E12.5 (Fig. 6E). GO analysis of the DEGs in cluster 1 revealed an enrichment of genes mainly involved in chemotaxis, suggesting that the migratory character of early PGCs is highly retained in *Dnd1*-cKO PGCs at E12.5 and down-regulated slowly to E13.5, compared with control PGCs. In contrast, the DEGs in cluster 2 were enriched for GO terms associated with multicellular organism development and cell differentiation, which included genes such as *Otx2*, *Sox3*, *Lefty1*, *Fgf5* and *Fgf15*. These genes are also known as markers for the primed state of pluripotency^34–36^, suggesting that PGCs may be inclined to become pluripotent ECCs in the absence of DND1.

On the contrary, down-regulated DEGs were also divided into two clusters　(Fig. 6F). GO analysis revealed that DEGs in cluster 3 were enriched for GO terms associated with the meiotic cell cycle, piRNA metabolic processes, and spermatogenesis. In contrast, GO terms were not significantly enriched in cluster 4. In the normal development of PGCs, the expression of meiotic genes such as *Sycp3*, *Syce2*, *Spo11*, *Meiob*, and *Meikin* gradually increases in both male and female PGCs after colonising embryonic gonads and is highly upregulated only in female PGCs after E14.5 (Supplementary Fig. S5A). However, male PGCs also show increased expression of these genes at relatively lower levels after E14.5 (Supplementary Fig. S5B), and subsequently begin to express genes involved in piRNA metabolic processes and spermatogenesis such as *Piwil1* and *Nanos2*^37^. Therefore, these data indicated that PGCs failed to enter the normal developmental pathway in male PGCs.

Collectively, these analyses support our hypothesis that in the absence of *Dnd1*, PGCs fail to undergo sexual differentiation, retain early PGC characteristics, and begin to enter the transformation pathway to ECC.

### Comparison of transcriptomic changes between *Dnd1*-cKO and *Ter* mutant PGCs

**Figure 7:**
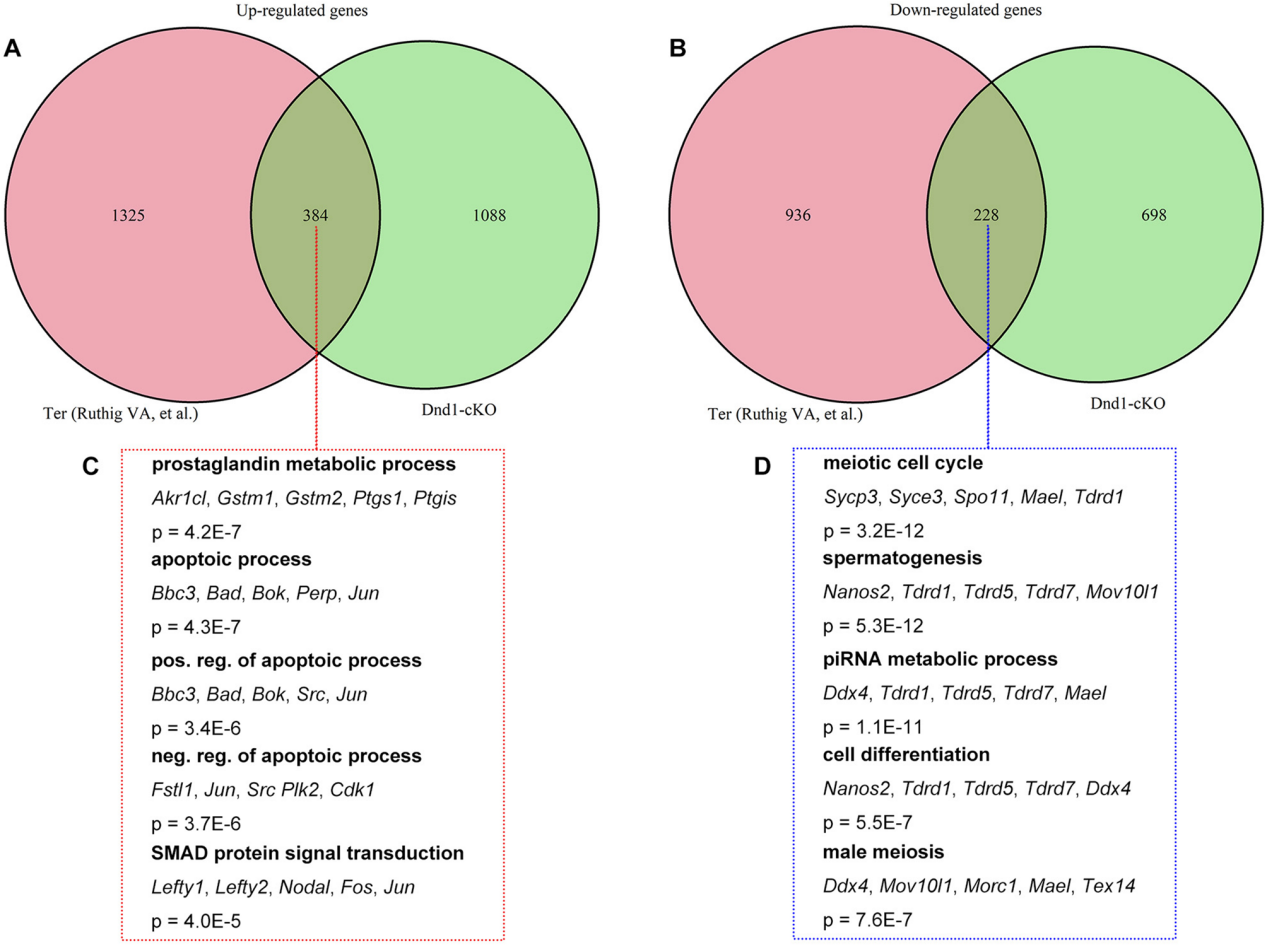
Comparison of transcriptomic changes between *Dnd1*-cKO and *Dnd1*^*Ter/Ter*^ PGCs. (**A**, **B**), The DEGs upregulated (**A**) and downregulated (**B**) in *Dnd1*-cKO PGCs at E12.5 and E13.5 are compared with those in *Dnd1*^*Ter/Ter*^ PGCs reported by Ruthig et al., respectively. (**C**, **D**), GO analysis of commonly upregulated (**C**) and downregulated (**D**) DEGs in both *Dnd1*-cKO and *Dnd1*^*Ter/Ter*^ PGCs.

Lastly, to further investigate the cause for transformation of ECC from PGC, we attempted to reveal common transcriptomic changes in *Dnd1*-cKO and *Ter* mutant PGCs. To this end, we extracted DEGs in the *Dnd1*^*Ter/Ter*^ PGCs at E12.5 and E13.5 from the RNA-seq results reported by Ruthig et al.^14^ using a threshold of 1.5 fold change as the cut-off value, and compared them with the DEGs in the *Dnd1*-cKO PGCs. These analyses identified 384 and 228 genes commonly up and downregulated in both *Dnd1*^*Ter/Ter*^ and *Dnd1*-cKO PGCs, respectively (Fig. 7A, B, and Supplementary Table S2). GO analysis revealed that the commonly downregulated genes were enriched for GO terms associated with the meiotic cell cycle, spermatogenesis, and piRNA metabolic process (Fig. 7C), which were consistent with the GO terms of downregulated DEGs in *Dnd1*-cKO PGCs, indicating that failure of sexual differentiation is a common feature of PGCs before the transformation to ECCs. On the other hand, the commonly upregulated genes were enriched for GO terms mainly associated with apoptosis, reflecting intensive apoptotic cell death in both mutant PGCs. In addition, we found a GO term associated with SMAD protein signal transduction (Fig. 7D), indicating that TGF-β superfamily signaling is active in both *Dnd1*^*Ter/Ter*^ and *Dnd1*-cKO PGCs immediately before transformation to ECCs. As a previous study reported that TGF-β superfamily signaling was also active in ECCs^13^, these results, therefore, raise a possibility that this signaling promotes the transformation of PGCs to ECCs.

## Discussion

In this study, we showed that deletion of *Dnd1* from migrating PGCs led to the accumulation of PGCs that failed to undergo sexual differentiation in the embryonic testes and found that ECCs were visibly developed from a portion of them after E16.5. Our transcriptomic analyses confirmed the failure of sexual differentiation and additionally revealed the upregulation of marker genes for the primed state of pluripotency, suggesting that PGCs are prone to becoming pluripotent ECCs in the testes of *Dnd1*-cKO embryos. In addition, we compared the DEGs in *Dnd1*-cKO PGCs with those in *Dnd1*^*Ter/Ter*^ PGCs and found enrichment of genes associated with a GO term of SMAD protein signal transduction in the commonly upregulated DEGs, suggesting the involvement of TGF-β signaling pathway in the ECC development. However, it remains unclear how only a portion of these PGCs is selected as ECCs. One possibility is that the interaction with specific tissue structures, such as basement membranes and mesonephros, and the paracrine factors from them would provide niches for PGCs to transform into ECCs. In this model, only PGCs in the niche can be transformed into ECCs. Alternatively, considering that ECC development is similar to that of induced pluripotent stem cells (iPSCs) in the context of dedifferentiation into a pluripotent state, there might be a subgroup of PGCs transcriptomically close to ECCs, from which ECCs might develop stochastically as well as iPSCs^38^. In either case, comparative transcriptomic analyses of PGCs transforming into ECCs and PGCs that do not transform into ECCs through the timing of ECC development would be an effective method for analysing the mechanism of ECC development. In this context, our *Dnd1*-cKO mouse line can be a useful tool for performing single-cell RNA sequence analyses of PGCs transformed into ECCs in the future.

We have previously shown that when *Dnd1* was deleted by tamoxifen injection at E13.5, PGCs failed to undergo male-type differentiation and were subsequently removed by apoptotic cell death, despite a comparable expression of NANOS2 with that of control PGCs^16^. This was probably due to NANOS2 dysfunction because DND1 is an essential partner for NANOS2 to regulate the male-type differentiation of PGCs in embryonic testes. Therefore, we presumed that even when *Dnd1* was removed from PGCs at migrating stages, small testes without germ cells could be observed due to NANOS2 dysfunction, as in the deletion of *Dnd1* at E13.5. However, we were surprised to find that PGCs transformed into ECCs and then generated teratomas in adult testes, which was never observed with the loss of *Dnd1* at E13.5. These data indicate that the deletion of *Dnd1* at the migrating stages makes PGCs more prone to transformation into ECCs than the deletion of *Dnd1* at E13.5. Given that ECCs are derived from PGCs that fail to undergo sexual differentiation, retaining the characteristics of migrating PGCs, the timing of *Dnd1* loss at E13.5 may be too late for transformation into ECCs since PGCs begin sexual differentiation by upregulating NANOS2 expression. In fact, it was recently reported that ECCs are derived from PGCs that do not express NANOS2 during embryonic testes^15^. Alternatively, the functions of DND1 in migrating PGCs may be different from those in PGCs that colonise the embryonic testes. Since only NANOS3, but not NANOS2, is expressed in migrating PGCs and DND1 is directly associated with NANOS3 in vitro^16^, NANOS3 may be a partner for DND1 in migrating PGCs. Therefore, the difference in target RNAs between the NANOS3-DND1 complex and the NANOS2-DND1 complex might generate the stage-dependent functional difference in DND1, resulting in susceptibility to STTs in the deletion of *Dnd1* at migrating stages. However, as DND1 is also implicated in mRNA protection from miRNA-mediated RNA degradation in other contexts, NANOS-independent DND1 function might play an important role in the suppression of STTs in migrating PGCs. Identification of RNAs associated with DND1 and DND1-partner protein in migrating PGCs is required to understand the mechanisms of transformation from PGC to ECC.

Mouse STTs occur predominantly on a genetic background of the 129 family of inbred mouse strains; although several kinds of gene mutation have been reported to increase the frequency of STT development in mice, STTs occurred only after introducing these mutations into a 129 strain genetic background by crossbreeding with 129 strain mice^5–9^. Therefore, genetic factors specific to the 129 strains, in addition to these gene mutations, is critical for the occurrence of STTs. However, the genetic factors specific to the 129 strains remains unidentified, making it difficult to analyse the mechanisms underlying the development of STTs on the 129 strain genetic background. In this study, we found that the conditional deletion of *Dnd1* in migrating PGCs caused STTs at a frequency of almost 100%, which was independent of the genetic factor of the 129 strains because the *Dnd1*-cKO mice were generated without 129 strain mice^9,16^. This result may indicate that STTs are solely caused by the loss of *Dnd1* in migrating PGCs. However, it is possible that the genetic background of the *Dnd1*-cKO mouse line itself has some factors that stimulate the generation of STTs. In fact, the ratio of the number of PGC in the cell cycle phase other than G0 (Fig. 4M, 24.01% in control) and the efficiency of EGC formation derived from PGC (Fig. 5H, 7.99% in control at E13.5) were both considerably higher than those previously described^31,39^. These data suggest that PGC development is delayed in this mouse line, leading to STT development at a high frequency^13^; however, we cannot exclude the possibility that the floxed alleles of *Dnd1* have some effect on the expression of DND1, thereby resulting in these phenomena. More critically, conventional deletion of *Dnd1* in the same genetic background of the *Dnd1*-cKO mouse line caused STTs, with a high probability of approximately 60% (Supplementary Fig. S6). This data indicates that some genetic factor stimulating STT development are present in the *Dnd1*-cKO mouse line, because C57BL/6J and MCH/ICR mice did not develop STTs or developed STTs at a very low frequency, even after the conventional deletion of *Dnd1*^9^. Therefore, unidentified genetic factors specific to the *Dnd1*-cKO mouse line might contribute to STT development, along with the deletion of *Dnd1* in migrating PGCs. To elucidate the effect of *Dnd1* loss on STT development in more detail, assays for STT development in the *Dnd1*-cKO mice should be performed on various genetic backgrounds. Alternatively, if other genetic factors are involved in the onset of STT in the *Dnd1*-cKO mouse line, identification of these factors using forward genetics should be considered to elucidate the mechanisms of STT development.

## Methods

### Animal experiments

All mouse experiments were conducted according to the protocol approved by the President of Yokohama National University after review by the Institutional Animal Care and Use Committee (Approval No. 2022-04). All methods were carried out in accordance with relevant guidelines and regulations. All methods are reported in accordance with ARRIVE guidelines.

### Mice

*Dnd1*-flox, *Oct4ΔPE-CreER*^*T2*^, and Rosa26-CreER^T2^ mice have been previously described^9,16^. In brief, the *Dnd1*_flox mouse line was established by using TT2 ES cells and maintained via intercrosses generating *Dnd1*^*flox/flox*^ mice, while the *Oct4ΔPE-CreER*^*T2*^ transgenic mouse line was generated by injecting the transgene into fertilised eggs of B6C3F1 mice and maintained by crossing with MCH/ICR mice. The Rosa26-CreER^T2^ mouse line was maintained on a genetic background of MCH/ICR strain.

The transgenic mouse line *CAG-CAT-EGFP*^40^ was provided by Dr. Junichi Miyazaki and maintained on a MCH/ICR genetic background, while *Nanog-GFP* transgenic mouse line^21^ was provided by RIKEN BRC through the National Bio-Resource Project of MEXT, Japan, on a mixed background of C57BL/6J and DBA, and subsequently crossed with the *Dnd1*^*flox/flox*^ mice. The PCR primer pair used for genotyping these lines was as follows: EGFP-Fw (5′-CTCGTGACCACCCTGACCTA-3′) and EGFP-Rv (5′-GTCCATGCCGAGAGTGATCC-3′).

### Tamoxifen treatment

Tamoxifen (T006000, Toronto Research Chemicals Inc., Canada) was dissolved in sesame oil (S3547, Sigma Aldrich, CA) at 20 mg/ml in a light-blocking tube at 55 °C. After filtration, the solution was administered to the pregnant mice in a single oral dose of 3 mg per mouse at E9.5 and E10.5, or 5 mg per mouse at E11.5 and E12.5.

### Haematoxylin and eosin staining

Testes were fixed with Bouin’s solution overnight, embedded in paraffin, and sectioned at 6 μm. After deparaffinisation, the sections were stained with haematoxylin and eosin according to standard procedures. Observations were performed under a microscope (AxioImager.M2; Carl Zeiss, Germany) equipped with a camera (Eos Kiss X2; Canon, Japan).

### Immunostaining

Embryonic testes were fixed with 4% paraformaldehyde overnight, embedded in paraffin, and sectioned at 4 µm. After deparaffinisation, the sections were autoclaved for 15 min at 105 °C with Antigen Unmasking Solution (H-3300, Vector Laboratories, CA) followed by blocking with 5% skim milk for 30 min, and then incubated overnight at 4 °C with primary antibodies against DAZL (1:2,000) produced by a guinea pig^16^, DND1 (1: 2,000) produced by a guinea pig^16^, NANOS2 (1:200) produced by a rabbit^22^, GFP (1: 1000; NB100-1770, Novus Biological, CO), NANOG (1:1000; IHC-00205, BETHYL Laboratories, MA), OCT4/POU5F1 (1:2000; sc-5279, Santa Cruz, TX), OTX2 (1:2,400; EPR20375, Abcam, UK), KI67 (1:2,000; RM-9106-S0, Thermo Fisher Scientific, MA), LAMININ (1:1,000; LAM-1, ICN Biomedicals, PA), cleaved CASPASE3 (1: 900; Asp175, Cell Signaling Technology, MA), or SYCP3 (1: 400; a gift from Dr. Chuma)^41^.

After washing with primary antibodies, the sections were incubated with donkey anti-guinea pig, anti-goat, anti-mouse, or anti-rat IgG conjugated with Aquamarine, Alexa488, or Alexa594 (1:500) (Jackson ImmunoResearch Laboratories Inc., PA, USA). Each section was counterstained with 0.5 μg/ml 4′,6-diamidino-2-phenylindole (DAPI) for 7 min, enclosed in Fluoromount™ (Diagnostic Biosystems Inc., CA), and then observed using fluorescence microscopy (AxioImager.M2; Carl Zeiss) and a CCD camera (AxioCam; Carl Zeiss). All antibodies were diluted with Can Get Signal Immunoreaction Enhancer Solution (Toyobo, Japan).

### Surveys for ECC colonies

Testes were excised from control and *Dnd1*-cKO embryos at E16.5, E17.5, and E18.5, and surveyed for ECC development by GFP fluorescence under a stereomicroscope (SZX16; Olympus, Japan) equipped with a CCD camera (AxioCam; Carl Zeiss). The incidence of ECC development was calculated as the percentage of male embryos in at least one ECC colony. Section immunostaining analysis using antibodies against GFP and NANOG was used to confirm ECC colonies that were ambiguous during the observation of whole testes.

### FACS analysis

Single-cell suspensions were prepared from the testes of embryos by incubation with Accutase (SF006, Merck Millipore, MA) at 37 °C for 40 min. After incubation, 10% newborn calf serum (S0750; Biowest, France) was added. After centrifugation at 1,800 rpm for 5 min, cells were re-suspended in sorting medium containing 2% newborn calf serum, 1% Glutamax (35,050,061, Thermo Fisher Scientific), 1% non-essential amino acids (11,140,050, Thermo Fisher Scientific), and DNA was labelled with 7-AAD (Beckman Coulter, CA, USA). FACS was performed using a Cell Sorter (MoFlo Astrios, Beckman Coulter, CA, USA).

### EGC culture

EGC was established as described previously^42^. Briefly, sorted PGCs were cultured on Mitomycin C (Kyowa Kirin, Japan)-inactivated Sl/Sl4-m220 feeder cells^43^ in DMEM (D6429, Sigma-Aldrich) supplemented with 15% KSR (10828010, Thermo Fisher Scientific), 1% Glutamax (35050061, Thermo Fisher Scientific), 1% non-essential amino acids (11140050, Thermo Fisher Scientific), 0.1 mM β-mercaptoethanol (198-15781, FUJIFILM Wako, Japan), 1000 unit/mL mLIF (ESG1107, Merck Millipore), and 12 ng/mL bFGF (450-33, PeproTech, NJ) until day 3. On day 3, the media was replaced with fresh media containing 1 μM PD325901 (04-0006, ReproCELL), 1 μM CHIR99021 (04-0004, ReproCELL, Japan) and 250 nM A83-01(039-24111, FUJIFILM Wako, Japan).

Cells were cultured under 20% O_2_ and 5% CO_2_ at 37 °C and the media were refreshed after 1, 3, and 5 days of culture. The efficiency of colony formation was determined after 7 days in culture as the number of colonies per seeded cell in a culture well.

### RNA-seq analysis

RNA-seq analyses were performed according to a method called Quartz-seq as previously described^33^. In brief, 100 PGCs from the testes of control and *Dnd1*-cKO embryos at E11.5 were directly sorted in a lysis buffer, and then reverse transcription (RT) was performed using Super Script III (12574018, Thermo Fisher Scientific) and an RT primer containing oligo-dT, T7 promoter, and PCR target region sequences. After removal and digestion of the RT primers using AMPure XP (A63880, Beckman Coulter) and exonuclease (2650, Takara Bio, Japan), a poly-A tail was added to the 3′ ends of the first-strand cDNA using terminal transferase (03289869103, Roche, Switzerland). Second-strand DNA was then synthesised using MightyAmp DNA polymerase (R076, Takara Bio) and a tagging primer. PCR amplification was performed using suppression primers. The amplified double-stranded cDNA was purified using a MinElute PCR Purification Kit (28004; Qiagen, Netherlands). Library preparation for RNA-seq was performed by ligation-based Illumina multiplex library preparation (LIMprep), as previously described^33^. Next-generation sequencing of cDNA libraries was performed using an Illumina HiSeq1500 (Illumina, CA).

Read files were mapped to the mouse reference genome (GRCm38 p6) using STAR aligner^44^ and only uniquely mapped reads were used for expression level estimation. Further analyses were conducted using R software (ver. 4.1.1). Gene counts were obtained using the ‘featureCounts’ function in the Rsubread package with ENSEMBL gene annotation (release98) and the genes showing log_2_(RPM + 1) values > 4 [greater than = 10–20 copies per cell]^45^ in at least one sample were used. The principal component analysis (PCA) was performed using the ‘prcomp’ function in the stats package (ver.3.6.2, https://rdocumentation.org/packages/stats/versions/3.6.2) with scaling. Gene ontology analysis was performed using DAVID as previously described^46^.

### Microarray analysis

Total RNA was extracted from sorted 120,000 PGCs in the testes of control and *Dnd1*-cKO embryos at E12.5 and E13.5, using the RNeasy Plus Micro kit (74034, Qiagen) according to the manufacturer’s instructions. The quality of the purified total RNA was verified using an Agilent 2100 Bioanalyzer (Agilent Technologies, CA). Conversion of isolated total RNA into cDNA and labelling of cDNA by cyanine-3 were performed using a Low Input Quick Amp Labelling Kit (One-Colour) (5190-2305, Agilent Technologies, CA). Labelled cDNA was fragmented, hybridised to SurePrint G3 Mouse GE 8 × 60 K v2 Microarray (Agilent Technologies) for 17 h at 65 °C and washed by using Gene Expression Hybridisation Kit and Gene Expression Wash Pack (Agilent Technologies). The microarray was scanned using Agilent SureScan G2600D, and the scan image data were analysed using Feature Extraction (11.5.1.1) software.

The raw gene expression data were processed using the limma package (ver. 3.28.14, https://www.rdocumentation.org/packages/limma/versions/3.28.14) for R (ver. 4.1.1). Briefly, raw data were first read using the ‘read.maimages’ function; then, background correction of microarray expression intensities and log2 transformation were performed using ‘backgroundCorrect’ and ‘normalizeBetweenArrays’ functions. After normalisation, control probes, probes with no gene symbols, and probes that were above the background on less than three arrays were filtered out. To perform gene-level interpretation, whole probes were collapsed into gene symbols by taking the maximum intensity among probe sets targeting the same gene. Differential gene expression analysis was performed using ‘lmFit’ and’ eBayes’ functions. Gene expression changes of > 1.5-fold and *p* value < 0.05 were considered significant. The principal component analysis (PCA) was performed using ‘prcomp’ function. GO analysis was conducted using DAVID, as previously described^46^ and GO terms with *p* values less than 1.0E-4 were considered significantly enriched. The heatmap was generated using the ‘heatmap.2’ function in the gplot package (ver.3.1.3, https://rdocumentation.org/packages/gplots/versions/3.1.3) after performing gene clustering using ‘dist’, ‘hclust’, and ‘cutree’ functions in the stats package. A Venn diagram were generated using the ‘venn.diagram’ function in the VennDiagram package (ver.1.7.3, https://www.rdocumentation.org/packages/VennDiagram/versions/1.7.3).

### Statistical analysis

The statistical significance of the differences in the ratios of cleaved CASPASE3-positive germ cells and KI67-positive germ cells and the efficiency of EGC formation was assessed using two-tailed *t*-tests. Statistical significance was set at *p* < 0.05. Statistical analyses were performed using Microsoft Excel.

## Supplementary Information

Supplementary Legends.

Supplementary Figure S1.

Supplementary Figure S2.

Supplementary Figure S3.

Supplementary Figure S4.

Supplementary Figure S5.

Supplementary Figure S6.

Supplementary Table S1.

Supplementary Table S2.

Supplementary Table S3.

## Data Availability

The datasets analysed during the current study are available in the GEO repository; https://www.ncbi.nlm.nih.gov/geo/query/acc.cgi?acc=GSE218770 for RNA-seq analysis, and https://www.ncbi.nlm.nih.gov/geo/query/acc.cgi?acc=GSE218744 for microarray analysis.
