# Supplementary material for: Loss of Dead end1 induces testicular teratomas from primordial germ cells that failed to undergo sexual differentiation in embryonic testes
Source: Sci Rep. 2023 Apr 19;13:6398. doi: 10.1038/s41598-023-33706-x (PMC10115811; doi:10.1038/s41598-023-33706-x)
Supplement: Supplementary file 1 — Supplementary Legends. [file 41598_2023_33706_MOESM1_ESM.docx]

***SUPPLEMENTARY INFORMATION***

**Loss of Dead end1 induces testicular teratomas from primordial germ cells that failed to undergo sexual differentiation in embryonic testes**

Atsuki Imai^1^, Kazuya Matsuda^1^, Yuki Niimi^1,#^, Atsushi Suzuki^1,2,*^

^1^Division of Materials Science and Chemical Engineering, Graduate School of Engineering, Yokohama National University, Yokohama, Kanagawa, Japan

^2^Division of Materials Science and Chemical Engineering, Faculty of Engineering, Yokohama National University, Yokohama, Kanagawa, Japan

^#^Current address: Center for Exploratory Research, Research & Development Group, Hitachi, Ltd., Kobe, Hyogo, Japan

^*^ Corresponding author:

E-mail: [suzuki-atsushi-gz@ynu.ac.jp](mailto:suzuki-atsushi-gz@ynu.ac.jp) (AS)

**SUPPLEMENTARY FIGURE LEGENDS**

**Supplementary Figure S1 | Quantification of phenotypes in *Dnd1*-cKO PGCs at E14.5, E15.5, E16.5, and E17.5.**

**A**, The number of DAZL-positive cells per area of a section was normalised by those of the corresponding control for each stage. More than 3 sections per embryonic testis were scored. *n*=3 biological replicates were quantified. **B**, Average NANOG signal intensity per PGC at E15.5 was normalised by that of control. More than 3 sections per embryonic testis were scored. *n*=3 biological replicates were quantified. **C**, The number of ECCs per area of section at E16.5 and E17.5 was normalised by that of E16.5. More than 3 sections per embryonic testis were scored. *n*=3 biological replicates were quantified. Black bars in **A**–**C** represent the mean ± SD. **p* < 0.05, ***p* < 0.01, ****p* < 0.001 (Student’s *t*-test).

**Supplementary Figure S2 | NANOS2 expression in E15.5 *Dnd1*-cKO testes.**

**A**–**D**, Testis sections from E15.5 *Dnd1*-cKO (*Dnd1^flox/flox^; Nanog-GFP; Rosa26-CreER^T2^*) embryos immunostained with antibodies against GFP (**A**), DND1 (**B**), and NANOS2 (**C**). A merged image is shown in **D**. **E**–**L**, Enlarged views of the area enclosed by the upper and bottom rectangles in **A**–**D** are shown in **E**–**H** and **I**–**L**, respectively. Images correspond to one of *n* = 3 biological replicates. Scale bars:100 μm.

**Supplementary Figure S3 | Gating strategy for FACS analysis.**

**A**–**N**, PGCs from testes (**C**–**H**) and ovary (**I**–**N**) in the control (*Dnd1^flox/flox^; Nanog-GFP*) (**C**–**E**, **I**–**K**) and *Dnd1*-cKO (*Dnd1^flox/flox^; Nanog-GFP; Rosa26-CreER^T2^*) (**F**­–**H**, **L**–**N**) embryos were gated based on size (FSC-H vs. SSC-H) (**A**), exclusion of cell doublets (FSC-A vs. FSC-H) (**B**), and EGFP signal and viability (EGFP vs. 7-AAD) (**C**–**N**).

**Supplementary Figure S4 | EGC derivation from PGCs in the ovary of the control and *Dnd1*-cKO embryos.**

**A**–**F**, Representative images of EGC colonies derived from EGFP-positive PGCs in the ovaries of control (*Dnd1^flox/flox^; Nanog-GFP*) (**A**–**C**) and *Dnd1*-cKO (*Dnd1^flox/flox^; Nanog-GFP; Rosa26-CreER^T2^*) (**D**–**F**) embryos at E13.5 (**A**, **D**), E14.5 (**B**, **E**), and E15.5 (**C**, **F**). EGC colonies were stained by alkaline phosphatase (AP) activity. Scale bars: 10 mm. **G**, Rate of EGC derivation from EGFP-positive PGCs in the ovary of the control (*Dnd1^flox/flox^; Nanog-GFP*) and *Dnd1*-cKO (*Dnd1^flox/flox^; Nanog-GFP; Rosa26-CreER^T2^*) embryos are indicated on the y-axis. PGCs were collected using FACS at the embryonic age indicated on the x-axis.

**Supplementary Figure S5 | Expression of meiotic genes in PGCs at E11.5, E12.5, E13.5, E14.5, and E15.5.**

**A**, **B**: Expression of *Sycp3*, *Syce3*, *Spo11*, *Meiob*, and *Meikin* in both male and female PGCs (**A**) and only in male PGCs (**B**). Pre-processed transcriptomes derived from male and female wild-type embryos^1^ were reanalysed using the pheatmap’ function of the pheatmap package (ver.1.0.12) in R (ver.4.1.3).

**Supplementary Figure S6 | Conventional deletion of *Dnd1* caused STTs in the same genetic background as the conditional deletion of *Dnd1*.**

**A**, Comparison of testes from 4-week-old wild-type, *Dnd1^+/∆^*, and *Dnd1^∆/∆^* mice with the same genetic background as *Dnd1^flox/flox^; Nanog-GFP; Rosa26-CreER^T2^* mice. Note that the *Dnd1^+/∆^* testis is smaller than that of wild-type as previously reported^2^ and left one of the two *Dnd1^∆/∆^* testes is affected with teratomas. Scale bar: 10 mm. **B**, Incidence of testicular teratomas in mice carrying the *Dnd1*-Δ allele generated from *Dnd1^flox/flox^; Nanog-GFP; Rosa26-CreER^T2^* mice. The number of male mice examined was as follows: wild type (n=47), *Dnd^+/∆^* (n=96), and *Dnd^∆/∆^* (n=51). Blue, orange, and green boxes indicate the percentages of cases in which the left, right, and both testes, respectively, were affected.

**SUPPLEMENTARY MATERIALS AND METHODS**

**Quantification of the number of DAZL-positive cells, NANOG signal intensity, and number of ECCs**

Embryonic testes were sectioned at 4 μm and immunostained with antibodies against DAZL, NANOG, and GFP. Then, more than three arbitrary sections per testis were selected at intervals of at least 20 μm to avoid counting the same cells, and images were acquired using fluorescence microscopy (AxioImager.M2; Carl Zeiss) and a CCD camera (AxioCam; Carl Zeiss) with aligned exposure times. The area of the sections were quantified by ImageJ software (version 1.50i).

The number of DAZL-positive cells was counted using the “Cell Counter” Plug-in in ImageJ software. Then, the value was corrected by the area of the section.

NANOG signal intensity was quantified as follows. First, NANOG signal intensity in each GFP-positive PGC was measured by “ROI Manager” tool in ImageJ software, and corrected by subtracting the background fluorescence intensity in the tubule to which each PGC belongs. Then, the values were averaged for each biological replicate.

For quantification of the number of ECCs, only NANOG-positive and DAZL-negative colony-forming cells were counted using the “Cell Counter” Plug-in in ImageJ software. Then, the value was corrected by the area of the section.

**Mice**

To establish a *Dnd1* conventional knockout mouse line with the same genetic background as *the Dnd1^flox/flox^; Nanog-GFP; Rosa26-CreER^T2^* mouse line, we crossed female *Dnd1^+/flox^; Nanog-GFP* mice with male *Dnd1^flox/flox^; Nanog-GFP; Rosa26-CreER^T2^* mice and obtained *Dnd1^+/flox^; Rosa26-CreER^T2^* female mice. After administering tamoxifen (75 mg/kg body weight), we crossed these female mice with male *Dnd1^+/flox^; Nanog-GFP* mice and obtained *Dnd1^+/∆^* offspring. The *Dnd1^+/∆^* mice were maintained by intercrossing.

**Tumour surveys**

Four-week-old male mice were surveyed for testicular teratomas. The incidence of teratoma was calculated as the percentage of male mice with at least one testicular teratoma. Histological analysis (haematoxylin and eosin staining) was performed to confirm any teratomas that were ambiguous at autopsy.

**SUPPLEMENTARY REFERENCES**

1. Miyauchi, H. *et al.* Bone morphogenetic protein and retinoic acid synergistically specify female germ-cell fate in mice. *EMBO J*. **36**, 3100-3119 (2017).

2. Imai, A. *et al.* Mouse dead end1 acts with Nanos2 and Nanos3 to regulate testicular teratoma incidence. *PLoS One*. **15**, e0232047 (2020).
