# Supplementary figures and images for: Loss of Dead end1 induces testicular teratomas from primordial germ cells that failed to undergo sexual differentiation in embryonic testes
Source: Sci Rep. 2023 Apr 19;13:6398. doi: 10.1038/s41598-023-33706-x (PMC10115811; doi:10.1038/s41598-023-33706-x)

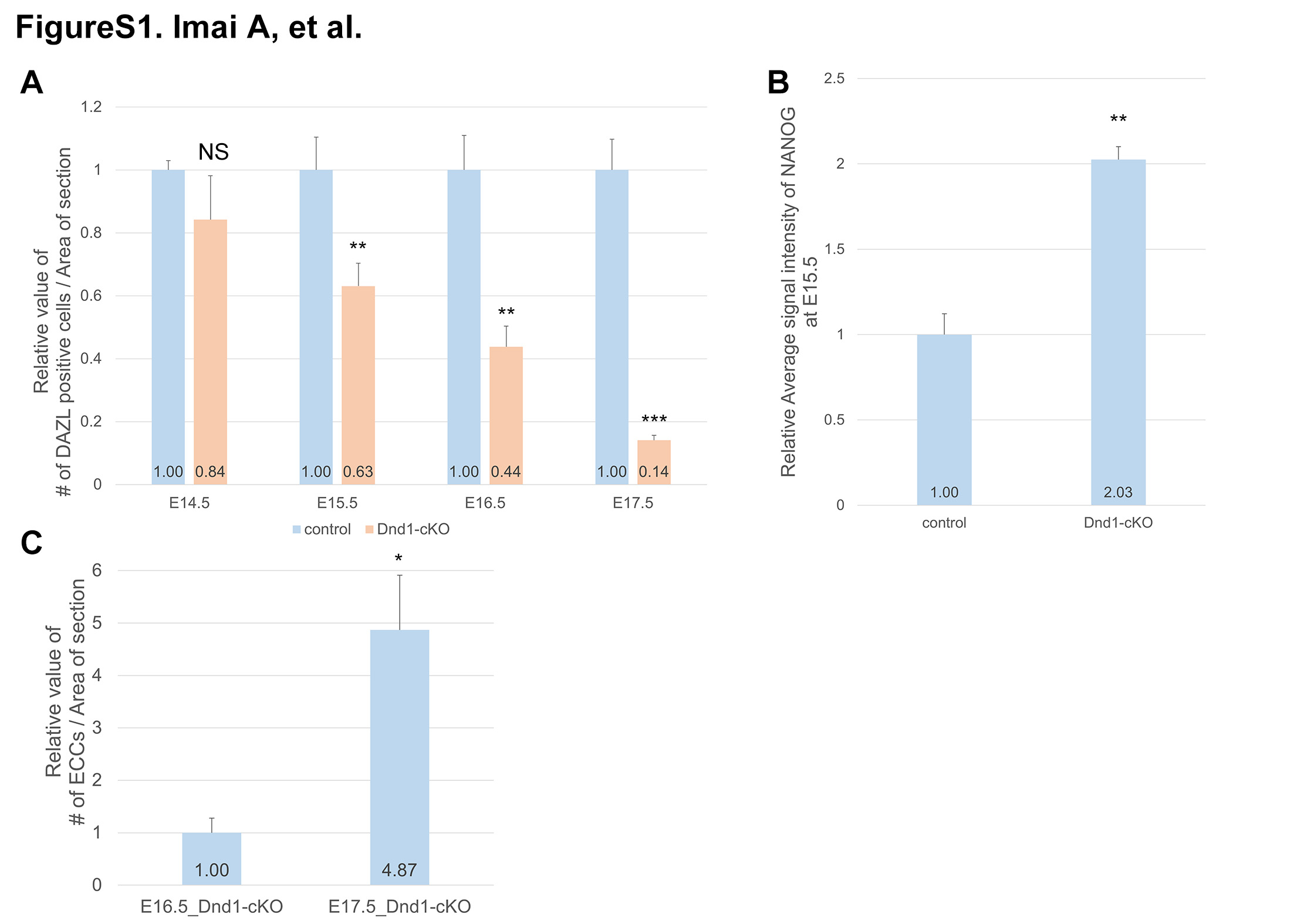

Supplement: Supplementary file 2 — Supplementary Figure S1. [file 41598_2023_33706_MOESM2_ESM.jpg]

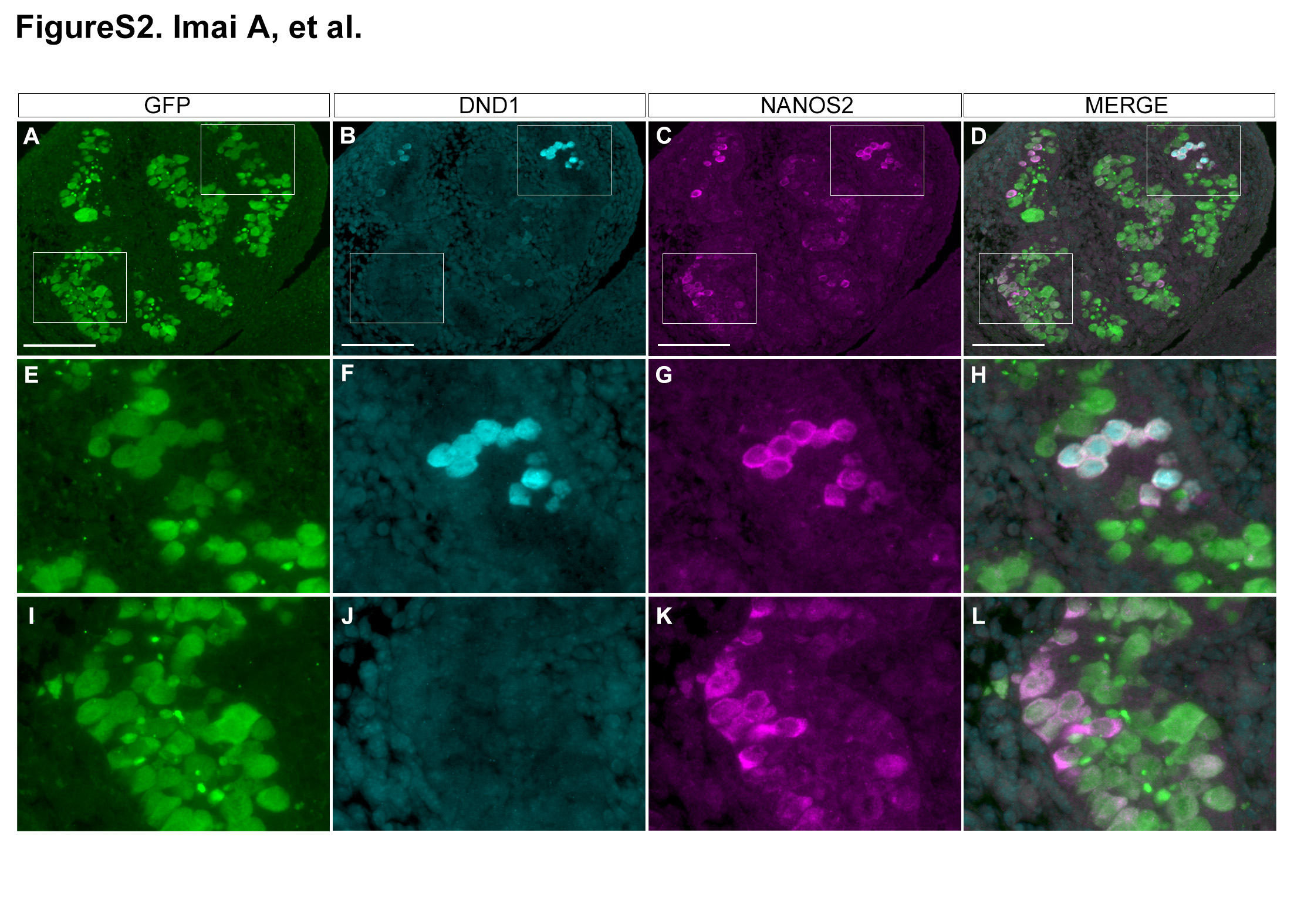

Supplement: Supplementary file 3 — Supplementary Figure S2. [file 41598_2023_33706_MOESM3_ESM.jpg]

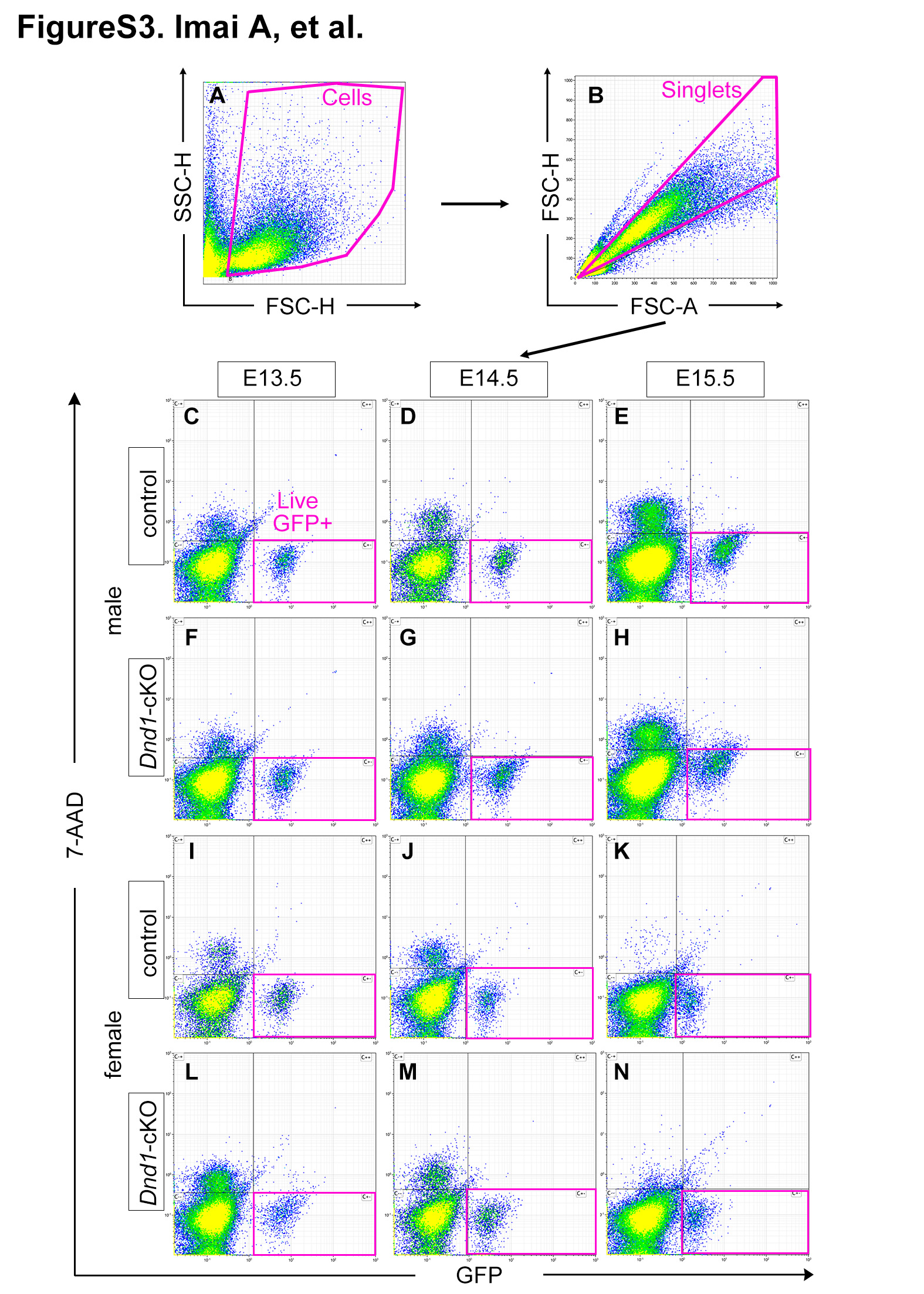

Supplement: Supplementary file 4 — Supplementary Figure S3. [file 41598_2023_33706_MOESM4_ESM.jpg]

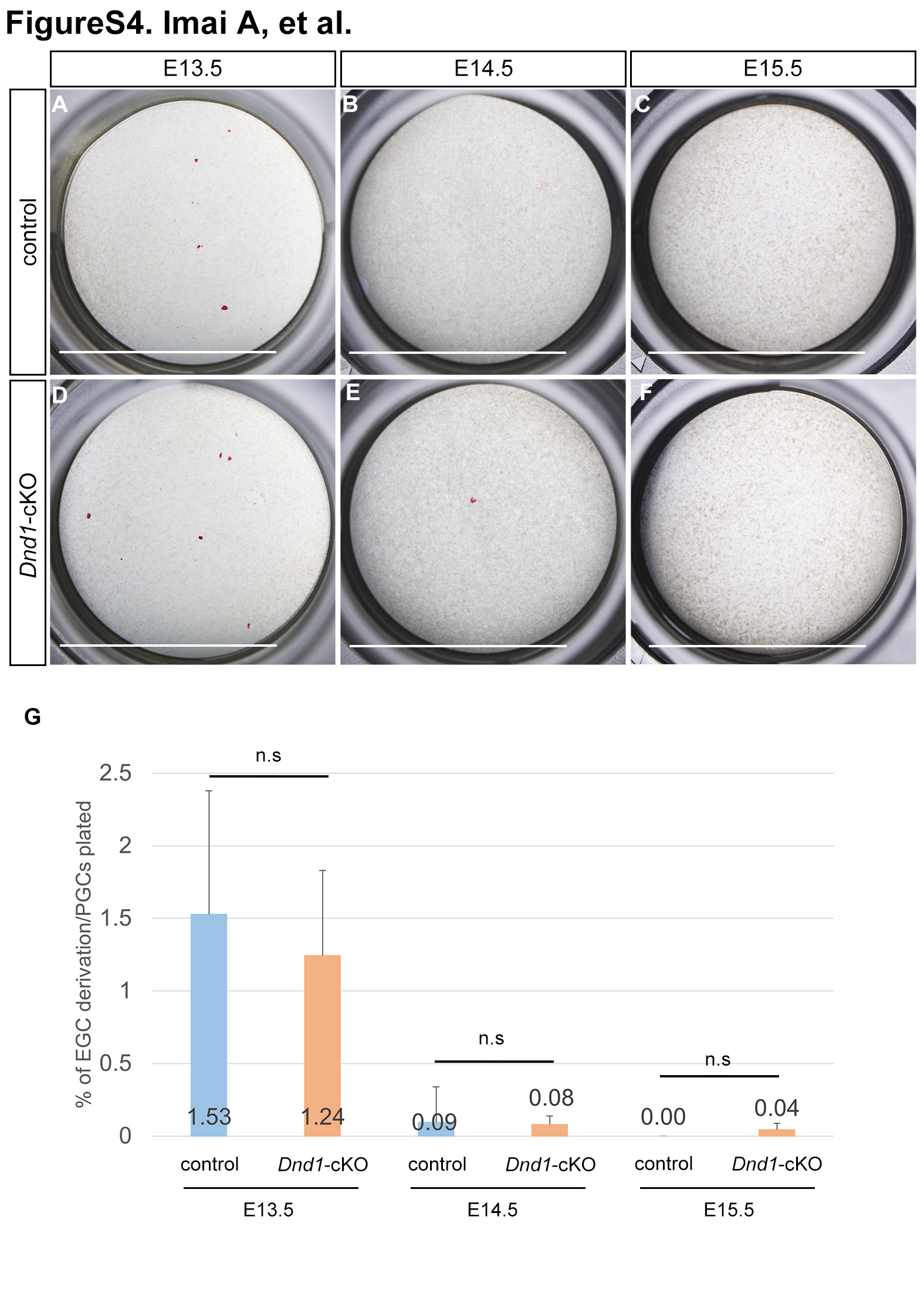

Supplement: Supplementary file 5 — Supplementary Figure S4. [file 41598_2023_33706_MOESM5_ESM.jpg]

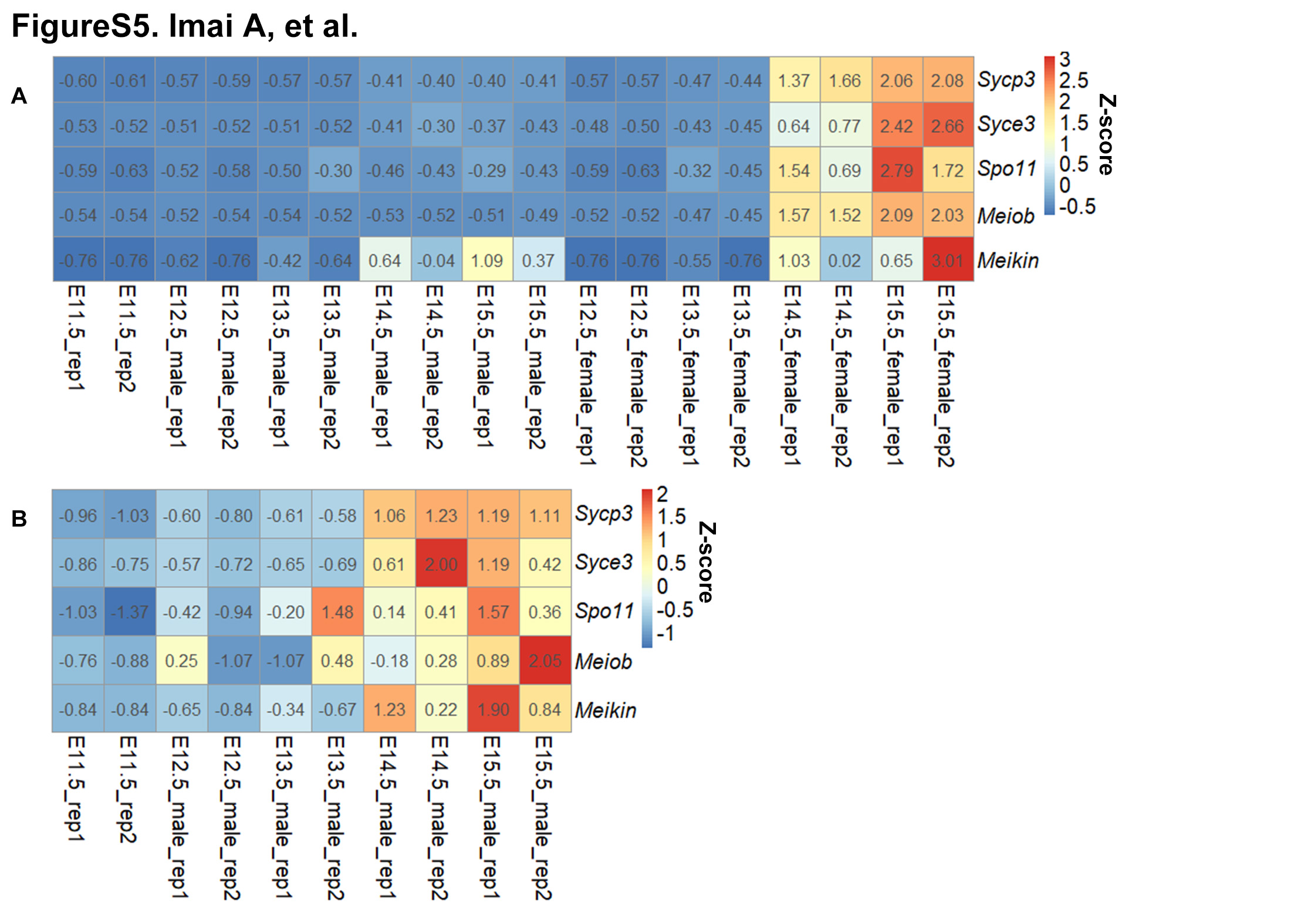

Supplement: Supplementary file 6 — Supplementary Figure S5. [file 41598_2023_33706_MOESM6_ESM.jpg]

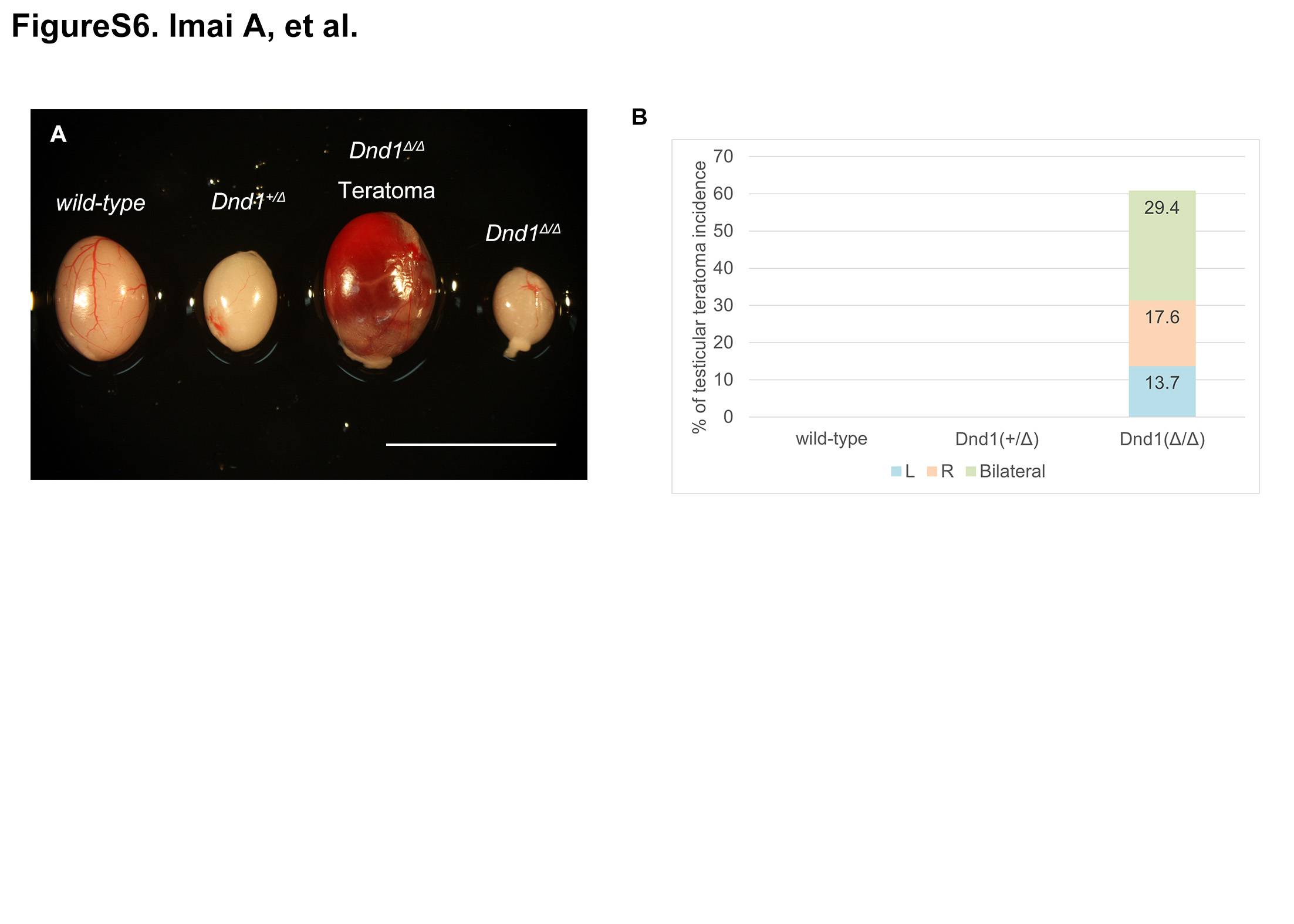

Supplement: Supplementary file 7 — Supplementary Figure S6. [file 41598_2023_33706_MOESM7_ESM.jpg]
